# Nanomaterials for Photothermal Antimicrobial Surfaces

**DOI:** 10.1021/acsomega.4c01449

**Published:** 2024-06-04

**Authors:** Lavinia Doveri, Yuri Antonio Diaz Fernandez, Giacomo Dacarro

**Affiliations:** † Department of Chemistry, University of Pavia, Via Taramelli 12, I-27100 Pavia, Italy; ‡ Centre for Health Technologies (CHT), University of Pavia, I-27100 Pavia, Italy

## Abstract

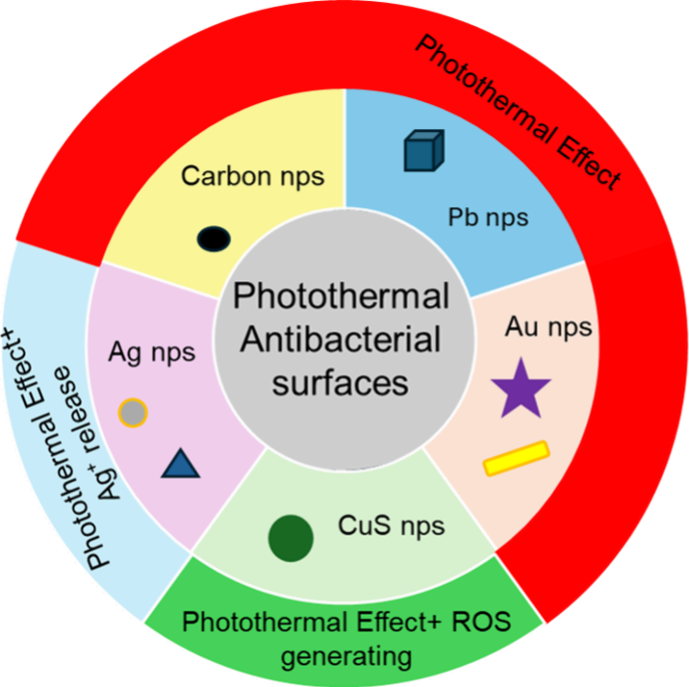

Microbial infection diseases are a major threat to human health and have become one of the main causes of mortality. The search for novel antimicrobial strategies is an important challenge for the scientific community, considering also the constant increase of antimicrobial resistance and the rise of new diseases. Among the new strategies to combat microbial infections, the photothermal effect seems to be one of the most promising. Hyperthermia is an effective and broad spectrum strategy for the removal of microbial infections. Among all of the strategies to reduce the diffusion of microbial infections, the preparation of antimicrobial surfaces seems of primary importance. In many cases, in fact, an infection can be diffused through surfaces just by touching them, or by inoculating microbes through an internalizable device, such as an implant, a prosthesis, or a catheter. In this review, we will summarize the recent advances in the preparation of photothermal antibacterial surfaces.

## Introduction

The search for protection from infections and diseases is not a recent idea. Even long before microbes were known to exist, there were strategies to prevent the diffusion of infections: in 800 BC Homer described the use of sulfur and fire to purify Odysseus’ house after the slaughter of the suitors. Silver vessels were used since the age of Alexander the Great to preserve food and water during military expeditions,^1^ while Romans used to put silver foil in their water for the same reason.^2^

Most of those methods of preventing infections relied on surfaces that could exert an antimicrobial effect. In more recent times, it became evident that antimicrobial materials are a fundamental means of defense against the spread of infections.

Microbial infection diseases are nowadays a major threat to human health and have become one of the main causes of mortality in the world. The search for novel antimicrobial strategies is an important challenge for the scientific community, considering also the constant increase of antimicrobial resistances and the rise of new diseases. Antimicrobial resistance (AMR) is a pressing global health concern that threatens the effective treatment of an ever-increasing range of infections caused by bacteria, parasites, viruses, and fungi. AMR occurs when microorganisms evolve mechanisms to withstand antimicrobial drugs, rendering standard treatments ineffective and leading to persistent infections, increased mortality rates, and the propagation of resistant strains. The rapid emergence and spread of AMR, driven by factors such as misuse and overuse of antimicrobials, inadequate infection control practices, and lack of new drug development, underscore the urgent need for concerted, multifaceted efforts to safeguard the efficacy of existing treatments and develop novel antimicrobial strategies. AMR is a recognized global health and development threat according to the World Health Organization (WHO), which declared AMR to be one of the top 10 global public threats facing humanity.^3^ In 2019, the six most common drug resistant bacteria alone (*S. aureus*, *S. pneumoniae*, *E. coli*, *K. pneumoniae*, *A. baumannii*, and *P. aeruginosa*) were estimated to have caused 929000 deaths.^4^ Viruses are a well-known threat as well: the recent COVID-19 pandemic was estimated to have caused a number of deaths ranging from 1.8 million to 3 million in 2020 only.^5^

Microbial infections can spread through several transmission mechanisms: infections can be transmitted through direct contact with infected individuals or their bodily fluids, such as saliva, blood, or respiratory droplets. Airborne transmission is common for respiratory viruses, while the fecal-oral route is a mode of transmission for both bacteria and viruses, often through contaminated food or water. Some infections spread through vectors such as mosquitoes, and others can be transmitted vertically from mother to child. Additionally, indirect contact with contaminated surfaces contributes to the spread of microbial infections. Understanding these transmission routes is crucial for implementing effective preventive measures, including vaccination, hygiene practices, and infection control protocols. Public health efforts aim to break the chain of transmission to minimize the spread of these infections.

Even when fomites (*i.e*. pathogen contaminated surfaces) are not the main cause of contagion (*e.g*. in the case of airborne viral infections), the spread of the infection through surfaces is always a relevant path of transmission. It has been reported that the number of contaminated surfaces can grow logistically under certain conditions, thus rapidly transmitting the infection.^6^ These problems become particularly relevant for healthcare associated infections (HAIs). These infections, acquired during the course of healthcare delivery, can result from various factors such as contaminated medical equipment, improper hygiene practices, and antibiotic-resistant organisms. HAIs not only compromise patient safety but also contribute to increased healthcare costs and longer hospital stays. Preventive measures, strict hygiene protocols, and antimicrobial stewardship are essential to mitigate the impact of HAIs and enhance overall healthcare quality.^7,8^ On any given day, about one in 31 hospital patients has at least one healthcare-associated infection, and The U.S. Department of Health and Human Services (HHS) has identified the reduction of HAIs as an Agency Priority Goal.^8^ There were an estimated 687,000 HAIs in U.S. acute care hospitals in 2015. About 72,000 hospital patients with HAIs died during their hospitalizations. In Europe, HAIs cause 16 million additional days of hospitalization each year, 37,000 attributable deaths, and 110,000 deaths for which infection is a contributing cause.^9^

Both viruses and bacteria can be transmitted through surfaces, but the former are less suited to survive and transmit the infection. The main difference between bacteria and viruses, in this regard, is that the former can grow in number on a surface, while the latter cannot. This is obviously due to the fact that viruses can multiply only with a host.

Some common strategies to avoid the transmission of microbial infections through surfaces are:

- Cleaning and disinfection of environmental surfaces: this is fundamental to reduce the potential contribution of contaminated surfaces to healthcare-associated infections. Cleaning and disinfection should follow standardized protocols and use appropriate products and technologies.^10,11^
- Hand hygiene and personal protective equipment: these are essential measures to prevent the transfer of microorganisms from contaminated surfaces to patients or healthcare personnel. Hand hygiene should be performed before and after contact with patients, their environment, or medical equipment. Gloves, gowns, masks, and other barriers should be used as indicated by the risk of exposure.^10,11^
- Antimicrobial surfaces: These are surfaces that have been modified to reduce or eliminate microbial growth or survival. They can be achieved by using materials with inherent antimicrobial properties, such as copper or silver, or by applying coatings or additives that confer antimicrobial activity. Antimicrobial surfaces can reduce the microbial burden on high-touch surfaces and potentially prevent cross-transmission.

Using the photothermal effect for the remediation and prevention of microbial infections is a promising approach for the development of antibacterial surfaces. This technique involves the use of materials that convert light energy into heat, effectively killing bacteria upon illumination. When these materials are exposed to near-infrared (NIR) light, they rapidly increase in temperature, leading to the destruction of bacterial cells. This method offers a nonantibiotic strategy for sterilization, presenting a broad-spectrum ability to combat bacteria without contributing to drug resistance.^12−14^ The versatility of photothermal materials allows for their integration into various applications, including in vitro and in vivo sterilization, solar water purification, and the creation of flexible antibacterial fabrics.^12^

In this review, we will summarize the state of the art and the recent advancements in the field of photothermal antimicrobial surfaces. Several recent reviews focused on the different kinds of photothermal nanomaterials^15,16^ or on a specific category,^12,17,18^ so we will leave to those publications the in-depth discussion of those topics. This paper will outline the applications of photothermal materials to the preparation of functionalized surfaces. We believe this is one of the most promising applications of photothermal nanomaterials with antimicrobial activity. Exploiting a photothermally active material anchored on a surface avoids all the concerns regarding the toxicity of a free nanomaterial that can accumulate in the body or release dangerous ions or molecules.

## Photothermal Antibacterial Effect

The fundamental principle behind the photothermal effect involves the utilization of materials, such as nanoparticles or nanomaterials, that possess the ability to absorb specific light wavelengths. Upon exposure to light of the appropriate wavelength, these materials efficiently convert light energy into heat, leading to a rapid increase in temperature in the vicinity of the target area. The mechanisms behind the photothermal effect can be different and depend on the nanomaterial’s composition. The main mechanisms of the photothermal effect have been recently described in an exhaustive review from Cui and co-workers.^15^

The photothermal conversion mechanism can be:

- A plasmonic localized heating caused by excitation and damping of a surface plasmon, typical of metal nanoparticles.
- A nonradiative relaxation of a semiconductor.
- Thermal relaxation of molecules due to lattice vibrations, common in carbon nanomaterials, polymers, and molecular materials.

The first materials studied in the literature for their photothermal effect were metals, with the first papers on this topic published in the 1980s. A good review of the studies that led to the biomedical use of photothermal nanomaterials can be found in a recent mini-review of Pallavicini et al.^16^ The photothermal effect has been widely exploited in the field of cancer treatment. After some seminal studies in the 1990s, using visible light irradiation and high laser powers, in the 2000s the synthesis of anisotropic gold nanoparticles opened the way to near-infrared (NIR) irradiation. Biological optical transparency occurs in the NIR region between 650 and 1800 nm, divided into the NIR-1 (650–950 nm), NIR-2 (100–1350 nm), and NIR-3 (1500–1800 nm) regions.^19,20^

The use of a laser wavelength in the so-called “bio-transparent window” allows irradiation of nanoparticles through tissues,^19^ and metal nanoparticles show high cross sections and photothermal conversions, allowing the use of low power lasers. The American National Standards Institute (ANSI) set the maximum permissible exposure for skin to 0.22 W/cm^2^ at 700 nm, 0.32 W/cm^2^ at 800 nm, and 1.0 W/cm^2^ in the range 1050–1400 nm.^21^ Having an effective hyperthermia using a laser irradiance below these standards is a fundamental prerequisite for any nanomaterial designed for through-tissue in vivo use. ICNIRP (International Commission On Non Ionizing Radiation Protection) guidelines recommend similar limits for laser exposure of skin: 0.328 W/cm^2^ for long exposure times (*i.e*. over 10 s) at 808 nm.^22^

Photothermal antibacterial materials have several advantages over traditional antibiotic therapies:^12,13,23,24^ they can achieve broad-spectrum sterilization against various types of bacteria, including drug-resistant ones,^25^ they can avoid the side effects of antibiotics,^26^ such as toxicity,^27^ allergy, and dysbiosis,^28^ they can be combined with other antibacterial strategies, such as photodynamic therapy, to enhance the synergistic effect, and they can be applied in diverse fields, such as in vitro and in vivo sterilization, solar water evaporation and purification, and flexible antibacterial fabrics.^29^

In the next sections, we will discuss the different materials that can be exploited for this purpose. Each section will be dedicated to a different material, based on chemical composition (*e.g*. gold, silver, etc.). When a significant number of papers is available for the same material, we will summarize in a table all the most relevant aspects of each work: *i.e.*, the laser irradiation conditions, the functionalization strategy, the substrate, etc.

We will also place a particular focus on the irradiation parameters: the magnitude of a photothermal effect is in fact strongly dependent on the laser wavelength and the irradiance. Moreover, high irradiances can generate striking effects but are not suitable for clinical use.

## Gold Nanomaterials

**Figure 1:**
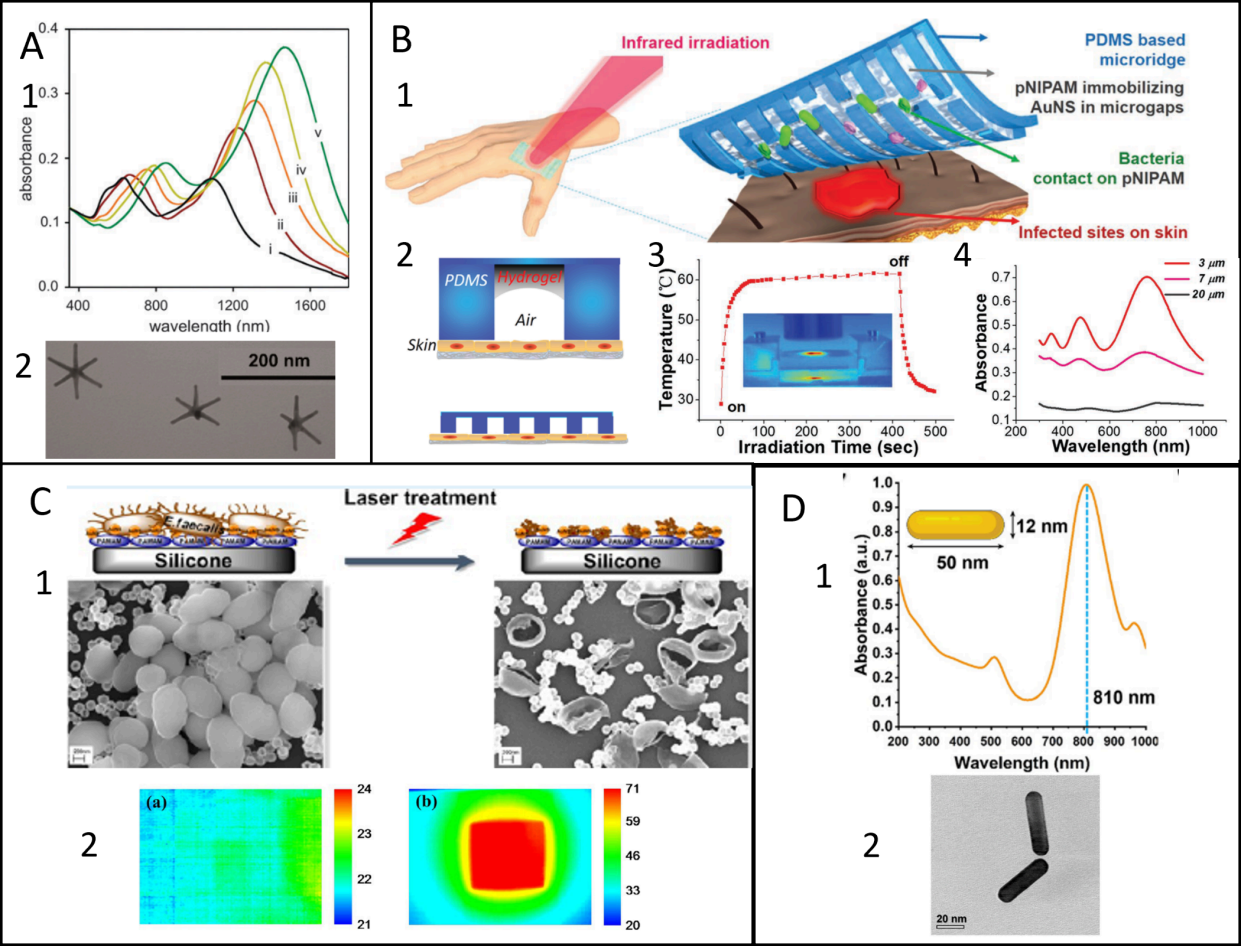
(A) 1. Absorption spectra of GNS obtained with increasing ascorbic acid concentration. Reproduced from ref (39) with permission from Royal Society of Chemistry (2013). 2. TEM image of GNS. Adapted from ref (43). Creative Commons Attribution 4.0 https://creativecommons.org/licenses/by/4.0/ (accessed on 12 February 2024). (B) 1. Functioning of TRIM photothermal films. 2. Sketch of the TRIM device. 3. Photothermal effect of TRIM film irradiated in the NIR. 4. UV–vis spectra of devices prepared with different amount of GNS. Adapted with permission from ref (51). Wiley (2020). (C) 1. SEM images of *E. faecalis* on AuNS modified PDMS before and after irradiation. 2. Thermal maps of bare PDMS (left) and AuNPS embedded in PDMS (right). Adapted with permission from ref (52). Copyright 2015 American Chemical Society. (D) 1. UV–vis spectrum of the Au NR solution. 2. High-res TEM image of two gold nanoparticles. Adapted from ref (61) with permission from Royal Society of Chemistry (2013).

Gold nanoparticles (AuNPs) are the most widely studied photothermal agents (PTAs) based on inorganic materials.^30−32^ Gold nanoparticles own peculiar electrical and optical properties, associated with high biocompatibility, that make them attractive for nanomedicine.^16,31,33,34^ As with other noble metal nanoparticles (Ag, Cu, and Pt),^35^ AuNPs possess a strong localized surface plasmon resonance band (LSPR), an interesting feature for photothermal issues. Plasmonic nanostructures are ideal candidates for light-to-heat conversion,^36^ since they can absorb incident photons with high cross-section and they convert them into heat through Landau damping of electron–hole pairs.^15^ These phenomena allow for an increase in local temperature on-demand, resulting in the possibility to kill bacteria (and microbes in general) with an external light stimulus. Moreover, a peculiar optical property of AuNPs, and plasmonic nanomaterials in general, consists of the possibility of fine-tuning the LSPR band position in the spectrum. In particular, it is possible to red-shift the band toward the NIR region, in the so-called “biotransparent window”, by simply changing size and shape. Common examples of anisotropic shapes for AuNPs are nanorods^37,38^ (*i.e*. cylindrical objects) and nanostars^39,40^ (multibranched objects).^16,41^ A summary of gold photothermal nanomaterials with specification about substrates, combination method, used laser, tested bacteria, and application is listed in Table 1. The synthesis of anisotropic AuNPs usually involves a seed growth method, and the growth is driven by the presence of a surfactant. Gold nanostars (GNSs), for example, can be obtained by using a zwitterionic surfactant (lauryl sulfobetaine, LSB) or a nonionic surfactant (Triton X-100), as reported in the literature.^39,42^ UV–vis spectra of GNSs, and anisotropic AuNPs in general, display multiple LSPR absorption bands: a weak transversal band near 500 nm and one or more intense longitudinal bands in the NIR, as shown in Figure 1A.^39^ GNSs can be grafted onto surfaces with a layer-by-layer (LbL) approach: GNSs are grafted on a thiol terminated monolayer, prepared by grafting a functional trialkoxysilane on a glass slide.^43^ Pallavicini et al.^44^ tested the photothermal antibacterial effect of these glasses when irradiated for 30 min at 808 nm with a 0.09W/cm^2^ irradiance (a value 4 times lower than the maximum allowed by ANSI), resulting in a reduction of *S. aureus* growth of 2 orders of magnitude. Using the same LbL technique, it is possible to grow a thin silica overlayer on GNSs, to increase mechanical and chemical stability. On top of this silica layer, a further layer of AgNPs can also be added: this imparts an intrinsic antibacterial effect to the surface, that can be enhanced in case of necessity by laser-triggered local hyperthermia.^45^ GNSs can also be grafted homogeneously onto an amino terminated monolayer on glass. These substrates showed a 99.99% reduction of Gram positive and Gram negative bacteria, when irradiated at 808 nm with a power density 0.265W/cm^2^ (within the safe limits for skin exposure by ANSI). Borzenkov et al.^46^ prepared poly vinyl alcohol (PVA) sprayed films with incorporated GNSs. Stability tests on multiple layers sprayed on glass surfaces established that the samples are stable in ambient conditions storage. However, the photothermal effect upon NIR (1024 nm with intensities within the safety level for medical applications) was moderate against *P. aeruginosa* strains (<54%).

**Table 1:** Summary of Photothermal Antibacterial Surfaces Based on Gold Nanomaterials

PTA	Substrate		Functionalization strategy	Antibacterial mechanism	Irradiation	Bacteria	Ref
Au nanostars	glass		electrostatic self-assembly	PTT	808 nm, 0.09 W/cm^2^	*S. Aureus*	(44)
			electrostatic self-assembly	PTT+Ag^+^	808 nm, 0.24 W/cm^2^	*S. aureus*, *E. coli*	(48)
			covalent bond	PTT	808 nm, 0.264 W/cm^2^	*S. aureus*, *E. coli*	(45)
			spray coating	PTT	850 nm; 0.35 W/cm^2^	*P. aeruginosa*, *S. aureus*	(46)
Au nanostars	titanium		electrostatic self-assembly	PTT	808 nm, 0.5 W/cm^2^	*E. coli*, *P. aeruginosa*, *S. aureus*, and *S. epidermidis*	(49)
Au nanostars	films	PVA	blending	PTT	1024 nm, 4 W/cm^2^	*E. coli*	(47)
		PDMS		PTT	808 nm, 0.24 W/cm^2^	*S. aureus*, *E. coli*	(43)
		PVA		PTT+Ag^+^	808 nm, 0.24 W/cm^2^	*S. aureus*, *E. coli*	(50)
Au nanostars	hydrogel	pNIPAM	blending	PTT	NIR, 0.7 1.5W/cm^2^	*S. aureus*	(51)
Au nanoshells	PDMS		covalent self-assembly	PTT	810 nm, 2.5 W/cm^2^	*E. faecalis*	(52)
AuNPs	silicon PDMS stainless steel		chemical platin method	PTT	810 nm, 2.3 W/cm^2^	*S. aureus*, *E. coli*	(53)
AuNPs@corn stalk	Chitin sponge		blending	PTT	808 nm, 2.0 W/cm^2^	*S. aureus*, *E. coli*,	(54)
Au nos@halloysite nanotubes	chitin hydrogel		blending	PTT	808 nm, 1.6 W/cm^2^	*S. aureus*, *E. coli*,	(55)
Au nanorods	hydrogel	chitosan	blending	PTT	810 nm, 0.2 W/cm^2^	*S. oralis*, *E. faecalis*	(56)
Au nanorods	polyurethane (PU)		blending	PTT	810 nm, 1.2 W/cm^2^	*P. aeruginosa*, *S. aureus*	(57)
			covalent self-assembly	PTT	808 nm, 0.8 W/cm^2^	*S. aureus*, *E. coli*,	(58)
Au nanorods	titanium		electrostatic self-assembly	PTT	808 nm, 1.5 W/cm^2^	*S. aureus*, *E. coli*,	(59)
Au nanorods	films	bacterial cellulose (BC)	blending	PTT	808 nm, 0.5 W/cm^2^	*S. aureus*, *E. coli*,	(60)
		electrospun PAN	electrospraying	PTT	NIR, 3.0 W/cm^2^	*S. aureus*, *E. coli*	(61)

With a similar approach GNSs were embedded in a PVA film.^47^ PVA films were also prepared with a mixture of GNSs and AgNPs,^50^ which proved to be effective against *E. coli* and *S. aureus* when irradiated in the NIR. The same method was used by Toci et al.,^43^ but using polydimethylsiloxane (PDMS) as a polymer increases the quantity of loadable GNSs and the substrates reach a final temperature of 65 °C, yielding a powerful on-demand photothermal antibacterial effect. All the presented functionalization strategies are suitable for coating medical devices like prostheses, catheters, implants, or wound dressings. The thermoablative approaches presented above can have macroscopic and systemic side effects. Hu et al.^51^ found an innovative and effective alternative called thermal-disrupting interface induced mitigation (TRIM), consisting in a localized thermal managing strategy to minimize the risk of skin damage during the heating process. The TRIM dressing films are designed as reported in Figure 1 B: the substrate alternates heat responsive regions and mechanically supportive regions. These wound dressing devices allow the temperature to reach 60 °C and are effective against *E.coli* and *P. aeruginosa* when irradiated for 30 min in the NIR spectrum with a high power lamp. The poly(*N*-isopropylacrylamide) coating used to distribute GNSs in substrate has a thermal response and also helps maintain the moisture of the skin.

Another promising photothermal tool has been presented by Khantamat et al.,^52^ which synthesized gold nanoshells (AuNSs) by surrounding silica nanoparticles with a thin layer of gold. These AuNSs are inert, and the LSPR band can be modulated by changing the dimensions of the dielectric core and of the gold shell. Carboxylic acid terminated AuNSs were grafted onto the surface of amino-terminated PDMS by a covalent bond. The photothermal effect was evaluated under irradiation of an 810 nm laser with a 2.5W/cm^2^ irradiance (10 times higher than the ANSI limits). The maximum temperature reached was 70 °C (Figure 1C.2), sufficient to exert a very powerful antibacterial effect against *E. faecalis*, as can be seen in Figure 1C.1. The chemical plating method can be used to deposit a thin layer of AuNPs (as photothermal agent) onto different types of flat surfaces, like silicon, PDMS, and stainless steel. These substrates are photothermally active against *E. coli* and *S. aureus* under 810 nm and 2.3W/cm^2^ irradiation.

AuNPs@corn stalk were embedded in chitin by Zeng et al.^54^ to prepare a photothermal antibacterial hemostatic sponge for wound healing applications. These materials show shape-memory properties and were found effective against *E. coli* and *S. aureus* under 808 nm irradiation, reaching high temperatures.

The aforementioned nanostars are not the only anisotropic shapes of AuNPs that can be prepared with straightforward synthetic procedures. Other promising shapes, with the same characteristics in terms of red-shift of the LSPR band, are cages and rods. Au nanocages (AuNCs) prepared by the galvanic replacement method were embedded in polyacrylamide-*co*-poly(acrylic anhydride-modified oxidized sodium alginate) (PAM-*co*-PAOSA) hydrogel, as shown in a paper from Chen et al.^69^ These films possess an absorption band at 800 nm that results in a local increase in temperature when irradiated, yielding a strong photothermal antibacterial effect against *E. coli* and *S. aureus* after irradiation. The last AuNP shape worth mentioning is gold nanorods (GNRs), which are widely used in the photothermal antibacterial field due to the two LSPR bands present in the UV–vis–NIR spectrum: one weak transverse band in the visible region and a second, more intense, longitudinal band centered in the NIR (Figure 1D.1). The seed growth synthesis to obtain GNRs typically involves the use of a cationic surfactant (cetyl trimethylammonium bromide).^16,61^ A representative TEM image is shown in Figure 1C.2. A metal broadly used in biomedical fields for prostheses is titanium. This material, however, does not own any antibacterial effect, and the implantation of a titanium prosthesis can lead to failure due to a biofilm infection. Yang et al.^49^ attached electrostatically negative GNRs on an amino-functionalized titanium surface. The LSPR of GNRs, centered at 800 nm, can be exploited to generate a local temperature increase that can effectively kill both *E. coli* and *S. aureus* after irradiation at 808 nm. The same authors also improved the efficiency of these functionalized surfaces by chelating zinc ions with polydopamine.^59^ In this way, the hyperthermia generated after irradiation enhances the release of zinc ions, adding a contact killing effect to the photothermal one.

Another clinical problem that can find interesting solutions with the use of nanomaterials is related to oral cavity infections, such as the formation of biofilms that lead to periodontal disease. Bermúdes-Jiménez et al.^56^ proposed a material made of a chitosan hydrogel with blended GNRs. The irradiation of these hydrogels with a laser centered at 810 nm and 0.2 W/cm^2^ irradiance allowed them to reach a temperature of 40 °C, sufficient to exert antibacterial activity against *S. oralis* and *E. faecalis*. GNRs were also bound on a polyurethane (PU) surface. PU is a versatile material, widely used in the biomedical field, for example for catheters. The first study conducted by Zhao et al.^57^ was focused on the functionalization of PU with (mercaptopropyl)-tryetoxysilane to allow covalent bonding with GNRs. The obtained photothermally active films in the NIR spectrum were further coated with thiol-modified PEG, an antifouling agent able to prevent the accumulation of bacterial debris. This approach was used by the same group to prepare safe hybrid nanocomposites with the addition of a quaternary ammonium salt.^58^ The irradiation of these GNRs-PU substrates at 808 nm with a lower power density with respect to the previous example (0.8 W/cm^2^ vs 1.2 W/cm^2^) results in a mild increase of temperature in a short time that enhances the effect of the quaternary ammonium salt that is an antibacterial agent.

GNRs can also be incorporated into films, like a bacterial cellulose one, by immersion to provide a photothermal antibacterial effect after NIR irradiation.^60^ Due to this evidence, these materials are suitable for wound dressing applications using environmentally friendly conditions with an on demand switchable enhanced antimicrobial effect. We have to stress the fact that all of the methods presented are easily applicable to various types of materials surfaces. After the COVID-19 pandemic, a significant issue arose regarding face masks, which have a short life and contribute to the biomedical waste, increasing cross contamination. A study conducted by Mohammad Ali Haghighat Bayan et al.^61^ proposed a straightforward method to prepare on-demand sterilizable innovative face masks with improved filtration properties. These innovative masks consist of a layer of GNRs electrosprayed between two layers of electrospun PAN nanofibers, to prevent Au release and improve filtration properties. The irradiation of these GNRs materials in the NIR allows sterilization and elimination of 99.95% of the bacteria on the surface due to the generated hyperthermia, making them reusable.

The toxicity of gold nanoparticles depends on size, dose, shape, and coating (*i.e.*, smaller NPs around 10 nm accumulate in many organs; surfactant increases the toxicity of AuNPs).^70−72^ However, in the studies presented, the AuNPs are not supposed to be released, resulting in biocompatible photothermal devices.

## Silver Nanomaterials

**Figure 2:**
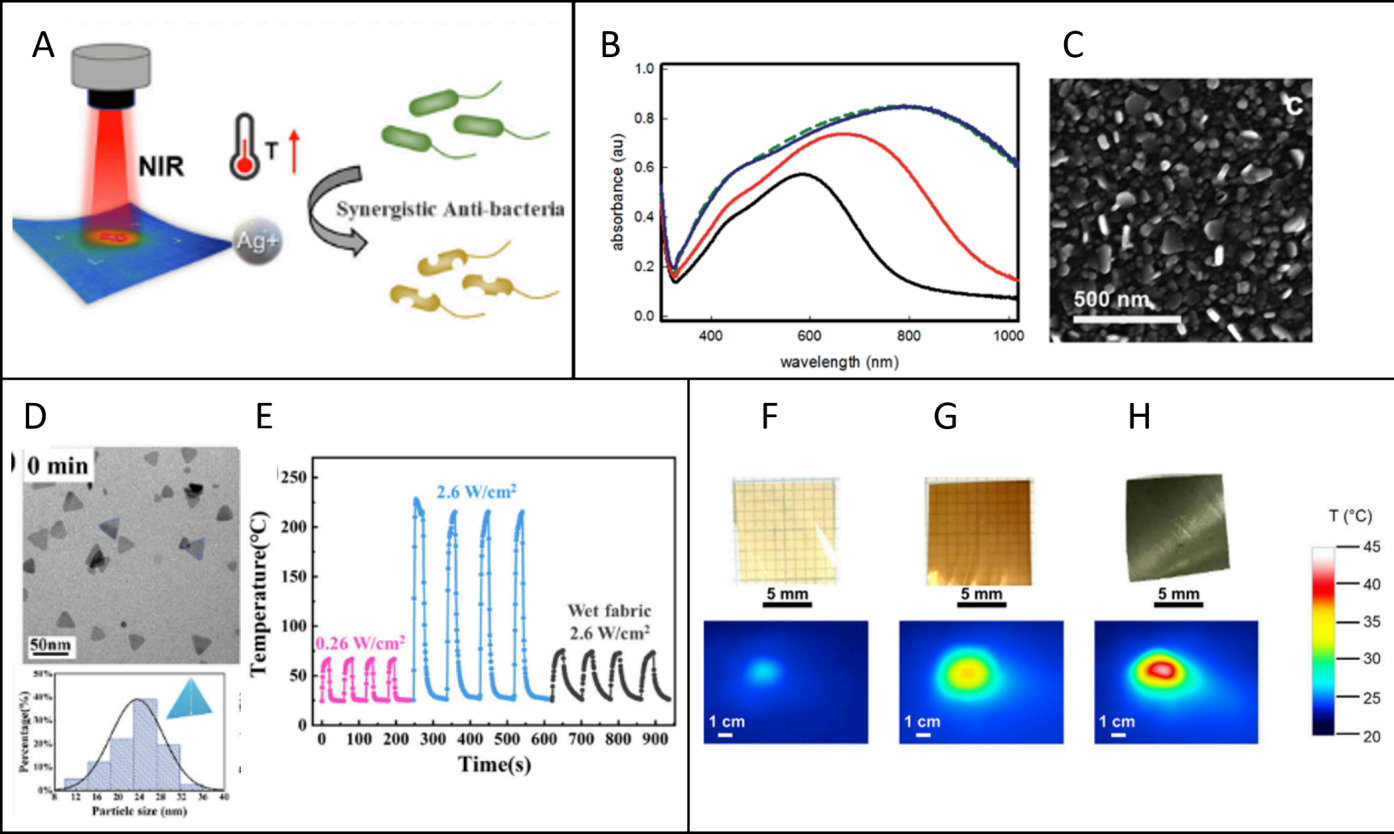
(A) Photothermal antibacterial mechanism of Ag nanomaterials. Reprinted from ref (77). Copyright 2022, with permission from Elsevier. (B) UV–Vis–NIR spectra of samples after different times of seed mediated growth: 15 min (black line), 30 min (red line), 2 h (blue line), 4 h (green dashed line) and (C) SEM images of 2 h samples. Adapted from ref (62) with permission from Royal Society of Chemistry. (D) TEM images of Ag nanotriangles and corresponding particle size distribution. Adapted from ref (77). Copyright 2022, with permission from Elsevier. (E) Temperature–time curve of blue Ag nanotriangles coated fabric repeated on–off cycles under 0.26 W/cm^2^ and 2.6 W/cm^2^ NIR irradiation and wetting state under 2.6 W/cm^2^ NIR irradiation. Adapted from ref (77). Copyright 2022, with permission from Elsevier. (F–H) Photographs and thermal maps of chitosan film (F), chitosan + lignin NPs (G), and chitosan + lignin@AgNPs (H). Reproduced from ref (78). Creative Commons Attribution 4.0 https://creativecommons.org/licenses/by/4.0/ (accessed on 12 February 2024).

The intrinsic bactericidal property of silver nanomaterials is well-known and largely reported in the literature.^73−76^ Cell death occurs due to the release of Ag^+^ ions and to the direct “nanomechanical” action of the high energy nanoparticles surface, which promotes the disruption of bacterial membranes. Spherical silver nanoparticles (AgNPs) synthesized with sodium borohydride and sodium citrate as capping and reducing agents typically show a LSPR band centered around 400 nm.^25,74,75^ The LSPR band, however, can be easily tuned and red-shifted to the NIR region just by changing the shape and size of the AgNPs.^77,79,80^ Using silver nanomaterials with a LSPR band centered in the NIR allows enhancement of the intrinsic antibacterial effect of silver with a switchable, on demand photothermal effect under NIR radiation^77,81^ (Figure 2A). The use of NIR light is also crucial for *in vivo* applications, since these wavelengths are in the biotransparent window, allowing direct irradiation through living tissue. In Table 2 silver photothermal nanomaterials are listed, specifying substrates, combination method, used laser, tested bacteria, and application. Anisotropic silver nanoplates can be grown directly onto PEI-functionalized glasses with a layer-by-layer (LbL) approach.^62^ Initially, a self-assembled monolayer of small-sized, spherical AgNPs was grafted. The substrates were then dipped in a growth solution containing Ag^+^/citrate/ascorbic acid, leading to the formation of Ag nanoplates with a broad and red-shifted LSPR band (Figure 2B). This is a good example of seed-growth synthesis supported on a substrate. The on-demand hyperthermia generated after irradiation (Δ*T* ≈ 60 °C) of the glasses with a laser at 808 nm 0.26 W/cm^2^ enhances the antibacterial effect against *S. aureus* and *E. coli* after only 30 min of irradiation. Moreover, this method results in the homogeneous coating of Ag nanoplates on glass surfaces as shown in Figure 2C. An alternative approach to prepare similar substrates consists in a two-step procedure: silver nanoplates can be prepared in colloidal form and then in a second step grafted onto PEI-functionalized glasses.^63^ In this case the glass surface was homogeneously coated with plates, but with a lesser coating density with respect to the previously mentioned paper. On the other hand, the shape of the nano-objects was more controlled. As a result, also the Δ*T* reached by these substrates was slightly lower (Δ*T* ≈ 35 °C) but still effective for photothermal antibacterial activity. These two approaches to functionalize bulk surfaces with silver nanoplates can be easily translated to prosthetic and subcutaneous devices (surgical sutures, for example), offering a long-term antibacterial effect reinforced on demand with laser-induced action. For the same purposes, triangular blue AgNPs (Figure 1 D) were prepared by a novel microwave assisted method and then impregnated on polyester/viscose blended spun lace nonwoven fabric.^77^ Thanks to the strong absorption in the NIR, the researchers evaluated the photothermal effect of these materials when irradiated with a laser centered at 808 nm with two power densities: 0.26 W/cm^2^ and 2.6 W/cm^2^, dried and wet. The results are displayed in Figure 2E, demonstrating that with low power density laser (below ANSI limits) the final temperature was ≈70 °C. With a high power laser in dry conditions, the temperature was so high that the sample burned, while high power in wet conditions led to a temperature comparable with that for the dry conditions irradiated with low laser power. AgNPs included in hydrogels are also reported in the literature as photothermal nanomaterials for wound healing.^64,65,67,68,82^ A study from Liu et al.^82^ described the use of gallic acid coated AgNPs (GA-AgNPs) as cross-linker for carrageenan films able to get Δ*T* ≈ 30 °C after irradiation with a 808 nm lamp with 2 W/cm^2^ irradiance. This material showed an antibacterial effect against Gram+ and Gram– bacteria. On the other hand the green colored hydrogel prepared by Li et al.^65^ contains mesoporous silica (MSN)/silver nanocomposites. The rod-like AgNPs formed in the channels formed by pores of mesoporous silica nanoparticles (MSN NPs) by mild reaction involving the reduction of Ag^+^ by butylamine that allows tuning the LSPR of the MSN/Ag nanocomposites in the NIR. Despite the fact that the LSPR of the gelatin hydrogel is in the NIR (600–900 nm), the authors investigated the photothermal conversion ability of the included MSN/AgNPs and the photothermal antibacterial effect only at 660 nm. A different strategy to tune the LSPR of silver nanoparticles from UV–vis to NIR was found by Merkl et al.^66^ by single step aerosol self-assembly. Plasmonic coupling among spherical AgNPs was controlled by the addition of a dielectric spacer (*i.e*. silica) that modulates the plasmonic interparticle distance. These Ag nanocomposites were used to coat and then encase poly dimethylsiloxane (PDMS), with tunable LSPR depending on the percent of AgNPs, with NIR properties and on demand ability to eradicate *E. coli* and *S. aureus* with 808 nm laser irradiation.

**Table 2:** Summary of Photothermal Antibacterial Surfaces Based on Silver Nanomaterials

PTA	Substrate		Functionalization strategy	Antibacterial mechanism	Irradiation	Bacteria	Ref
Au nanostars	glass		electrostatic self-assembly	PTT	808 nm, 0.09 W/cm^2^	*S. aureus*	(44)
			electrostatic self-assembly	PTT+Ag^+^	808 nm, 0.24 W/cm^2^	*S. aureus*, *E. coli*	(48)
			covalent bond	PTT	808 nm, 0.264 W/cm^2^	*S. aureus*, *E. coli*	(45)
			spray coating	PTT	850 nm, 0.35 W/cm^2^	*P. aeruginosa*, *S. aureus*	(46)
Au nanostars	titanium		electrostatic self-assembly	PTT	808 nm, 0.5W/cm^2^	*E. coli*, *P. aeruginosa*, *S. aureus*, and *S. epidermidis*	(49)
Au nanostars	films	PVA	blending	PTT	1024 nm, 4 W/cm^2^	*E. coli*	(47)
		PDMS		PTT	808 nm, 0.24 W/cm^2^	*S. aureus*, *E. coli*	(43)
		PVA		PTT+Ag^+^	808 nm, 0.24 W/cm^2^	*S. aureus*, *E. coli*	(50)
Au nanostars	hydrogel	pNIPAM	blending	PTT	NIR, 0.7–1.5 W/cm^2^	*S. aureus*	(51)
Au nanoshells	PDMS		covalent self-assembly	PTT	810 nm, 2.5 W/cm^2^	*E. faecalis*	(52)
AuNPs	silicon PDMS stainless steel		chemical platin method	PTT	810 nm, 2.3 W/cm^2^	*S. aureus*, *E. coli*	(53)
AuNPs@corn stalk	Chitin sponge		blending	PTT	808 nm, 2.0 W/cm^2^	*S. aureus*, *E. coli*	(54)
AuNPs@halloysite nanotubes	chitin hydrogel		blending	PTT	808 nm 1.6 W/cm^2^	*S. aureus*, *E. coli*	(55)
Au nanorods	hydrogel	chitosan	blending	PTT	810 nm, 0.2 W/cm^2^	*S. oralis*, *E. faecalis*	(56)
Au nanorods	polyurethane (PU)		blending	PTT	810 nm, 1.2 W/cm^2^	*P. aeruginosa*, *S. aureus*	(57)
			covalent self-assembly	PTT	808 nm, 0.8 W/cm^2^	*S. aureus*, *E. coli*,	(58)
Au nanorods	titanium		electrostatic self-assembly	PTT	808 nm, 1.5 W/cm^2^	*S. aureus*, *E. coli*,	(59)
Au nanorods	films	bacterial cellulose (BC)	blending	PTT	808 nm, 0.5 W/cm^2^	*S. aureus*, *E. coli*,	(60)
		electrospun PAN	electrospraying	PTT	NIR, 3.0 W/cm^2^	*S. aureus*, *E. coli*	(61)

The last example of this section regards chitosan films embedded with Ag@lignin NPs that are able to absorb 89% of the radiation across the entire solar spectrum. This evidence allows increasing temperature up to 51 °C in only 2 min after simulated solar radiation at 0.1 W/cm^2^ (Figure 2F–H). This solar activated nanomaterial exhibits a significant switchable photothermal antibacterial effect against *E. coli*, offering an interesting alternative to the classic laser irradiation.^78^ The use of AgNPs widespread in household products related to everyday life. Their cytotoxicity is size-dependent, like happens for AuNPs, along with the effect of ion release that generates ROS incrementing the toxicity.^70,83^

## Copper Chalcogenides Nanomaterials

**Figure 3:**
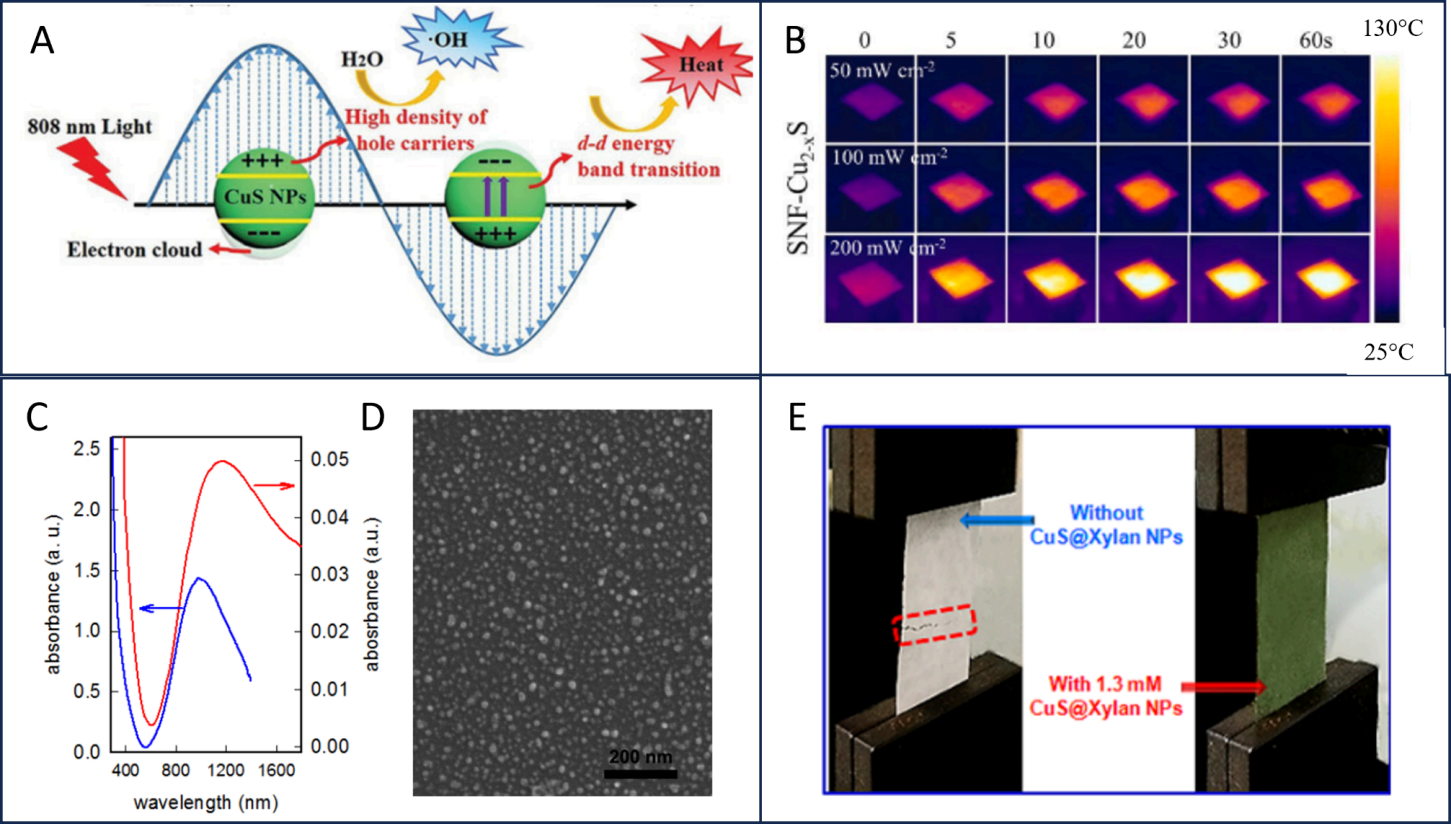
(A) Photothermal and photodynamic processes in copper chalcogenides under NIR light. Adapted from ref (92), with permission from Royal Society of Chemistry (2018). (B) Thermographic images of SNF-Cu_2–*x*_’s under different power densities. Reprinted from ref (103), with permission from Elsevier. (C) UV–vis spectrum of a CuS NP-glass sample (red line) compared with the spectrum of the colloidal suspension used for functionalization (blue line) and (D) SEM image of the CuS NP-glass sample. Adapted from ref (87). Creative Commons Attribution 4.0 https://creativecommons.org/licenses/by/4.0/ (accessed on 12 February 2024). (E) Tensile test of composite paper and CuS@Xylane functionalized paper. Adapted with permission from ref (94). Copyright 2017 American Chemical Society.

In the past decade the attention of researchers has been attracted by copper chalcogenides because of their good biocompatibility and effective photothermal conversion.^84,85^ These characteristics make them perfect candidates for application as photothermal antibacterial agents.^18,86,87^ Binary copper chalcogenides with the generic formula Cu_(2–*x*)_E (E = S, Se, Te and 0 < *x* < 1) are formed by covalent bonds (rather than ionic) with selenium, sulfur, and tellurium. Their atomic ratio and structure depend on the oxidation state of copper.^18^ Copper chalcogenides show very strong local surface plasmon resonance (LSPR) from near-infrared (NIR) to mid-infrared (MIR) due to copper holes in the valence band that oscillate, resulting in strong absorption and scattering of incident light. On the other hand, Cu_2_S (the full stoichiometric compound), is LSPR inactive because of the absence of free holes. LSPR in copper chalcogenides has an important difference with LSPR in noble metal nanoparticles: in the case of noble metal NPs, the LSPR is affected by the size, while for chalcogenides, LSPR is strongly dependent on the degree of copper deficiency (*i.e.*, atomic ratio to copper chalcogenide).^18,86−88^ As for noble metal NPs, copper chalcogenides can efficiently convert NIR light into heat for photothermal therapy.^18,30,85,87^ Among all the existing copper chalcogenides, copper sulfides (Cu_(2–*x*)_S) are the most studied due to their semiconducting properties and their possible application in several different fields (*e.g*. catalysis,^89^ photovoltaic,^90^ photothermic,^91^*etc.*). More in detail, Cu_(2–*x*)_S NPs are p-type semiconductors with an LSPR band generated by collective oscillation of their holes that results in thermal relaxation when irradiated. The position of the LSPR band can be tuned by changing the number of carriers, *i.e*. modifying the stoichiometry.^85,87,88^ In Table 3, copper chalcogenide photothermal antibacterial materials are enlisted, specifying substrates, combination method, used laser, tested bacteria, and application. In general, it is possible to notice that in all the cases the antibacterial mechanism is a combination of photothermal therapy (PTT) and photodynamic therapy (PDT), as depicted in the scheme in Figure 3A. When irradiated in the NIR, copper chalcogenides generate hyperthermia and are also able to generate reactive oxygen species (ROS), which can damage DNA, RNA, and proteins.^13,18,87,92^ A promising use of copper sulfide nanoparticles was exploited by Ren et al.:^93^ photothermal Cu_(2–*x*)_S NPs were used to decorate surgical face masks. These novel cost-effective nanomaterials undergo photothermal sterilization in a very short time when irradiated by an IR lamp. More in detail, Cu_(2–*x*)_S NPs were directly synthesized on the surface of spun-bonded nonwoven fabric (SNF) layers from surgical masks. The surface of the functionalized mask rapidly heats to 78 °C when irradiated in the NIR with 50 mW/cm^2^ irradiance (Figure 3B), thanks to the ability of Cu_(2–*x*)_S NPs to convert IR energy into heat. Hyperthermia enables thermal ablation of Gram+ and Gram– bacteria and grants inactivation of human coronavirus OC43 and influenza A virus A/PR/8/34 (H1N1). Photothermal antibacterial textiles for preventing antibacterial infection in everyday life are proposed by Ren et al.^93^ by chelating CuS NPs on polyaniline (PANI) functionalized silk fabric. PANI is a chelator agent used to bind copper to the silk surface, obtaining a stable and photothermal surface able to efficiently kill *S. aureus* and *E. coli* after only 5 min of irradiation with an IR lamp (200 mW/cm^2^), thanks to the increased temperature of the surface and the generation of ROS.

**Table 3:** Summary of Photothermal Antibacterial Surfaces Based on Copper Chalcogenides Nanomaterials

PTA	Substrate		Functionalization strategy	Antibacterial mechanism	Irradiation	Bacteria	Ref
CuS NPs	paper		hydrogen bonding	PTT+PTD	808 nm, 1.5 W/cm^2^	*S. aureus, E. coli,B. subtilis*, A. niger	(94)
CuS NPs	hydrogel	MES	blending	PTT+PTD	808 nm, 2 W/cm^2^	*S. aureus, E. coli*,	(92)
		Gel-MA		PTT+PTD	808 nm, 1.2 W/cm^2^	*S. aureus, E. coli*	(95)
		Guar gum		PTT	660 nm, 1.5 W/cm^2^	*E. coli*	(96)
		PVA		PTT+PTD	808 nm, 1.3 W/cm^2^	*S. aureus, E. coli*	(97)
		Modified HA		PTT	980 nm, 1.5 W/cm^2^	*S. aureus, E. coli*	(98)
CuS NPs	glass		LbL-electrostatic interaction	PTT+PTD	950 nm, 0.35 W/cm^2^	*P. aeruginosa*	(87)
CuSe	PVDF		blending	PTT+PTD	1064 nm, 3 W/cm^2^	*S. aureus, B. subtilis*	(99)
CuS NPs	electrospun fibers	chitosan	blending	PTT	980 nm, 1 W/cm^2^	*no*	(100)
		chitosan	blending	PTT	980 nm, 1.5 W/cm^2^	*no*	(101)
Au@CuS NPs	PDMS		blending	PTT+PTD	808 nm, 3.5 W/cm^2^	*E. coli*	(102)
Cu_2–*x*_S	spun-bonded nonwoven fabrics (SNF) coupled		in situ- growth	PTT+PTD	IR, 0.5–2 W/cm^2^	*E. coli, S. aureus HCoV-OC43, PR8*	(103)
CuS NPs	silk fabric		chelation	PTT+PTD	300–2500 nm, 0.2 W/cm^2^ (simulated daylight lamp)	*E. coli, S. aureus*	(104)
Ti_3_C_2_@CuS	polyurethane		blending	PTT+PTD	simulated daylight lamp	*no*	(105)
CuS@g-C_3_N_4_ nanoparticle	scaffolds	poly-l-lactide	blending/selective laser sintering	PTT+PTD	808 nm, 1 W/cm^2^	*E. coli, S. aureus*	(106)

A two-step method for bulk (glass) surfaces functionalization with CuS NPs has been proposed by Gargioni et al.^87^ CuS NPs were synthesized in aqueous media and then grafted onto glasses by electrostatic bond with a straightforward method involving positively charged trialkoxy-silanes. When NPs are grafted on glass, the LSPR band of the 5–20 nm CuS NPs shifts from 900 to 1250 nm, as shown in Figure 3C, and a homogeneous and densely packed layer of NPs is obtained (Figure 3D). Activation of the photothermal effect on these layers with a 950 nm laser and 0.35 W/cm^2^ irradiance (below ANSI limits) leads to successful *P. aeruginosa* eradication.

Usually CuS materials show an intrinsic antibacterial activity, due to the presence of copper ions. This can be strongly enhanced by the photothermal action, which is switchable on demand through NIR light irradiation. Some examples regarding the use of CuS NPs to produce hydrogels for wound healing exist in the literature. In all the cases CuS NPs are blended in different matrixes such as 3-(trimethoxysilyl)propyl methacrylate (MES),^92^ methacrylate-modified gelatin (Gel-MA),^95^ modified hyaluronic acid (HA),^98^ guar gum,^96^ and poly vinyl alchol (PVA).^97^ The photothermal effect was evaluated under irradiation of a lamp at 808 nm with different power densities (2 W/cm^2^,^92^ 1.2 W/cm^2^,^95^ 1.3 W/cm^2,97^). These substrates show a triple mode synergistic effect of photothermal, photodynamic, and peroxidase like activity to eradicate bacteria and biofilm while promoting the wound healing process when irradiated in the NIR.^92,95,97^ The influence of the shape of CuS NPs embedded in chitosan fibers on the photothermal response under irradiation at 980 nm and 1 W/cm^2^ was investigated by Cheng et al.^100^ The plate-like CuS NPs allow reaching 53 °C in 90 s, that is 10 °C higher with respect to tube-like, flower-like, and sphere. Moreover, this group evaluated the interaction between CuS NPs and chitosan by changing the coating with various interfaces (xylan, sodium alginate, poly ethylene glycol). As a result, xylan coated CuS NPs improved not only the mechanical properties but also the photothermal response, reaching ≈60 °C in 90 s with a 980 nm laser.^101^ A NIR light triggered antimicrobial paper has been developed by Huang et al.^94^ by placing CuS@Xylan NPs of 10 nm of diameter in cellulose fibers (CNFs). The resulting paper has the typical green color of CuS NPs (Figure 3E) and shows improved mechanical properties with respect to plain paper, due to the strong hydrogen bonding between xylan and CNFs. When irradiated with a 808 nm and 1.5 W/cm^2^ laser for 2 min, these materials reach ≈70 °C, resulting in a high photothermal antibacterial effect against *S. aureus*, *E. coli*, *B. subtilis*, and *A. niger*. Studies regarding the toxicity of CuS NPs demonstrated that these nanoparticles are not cytotoxic to human dermal fibroblast cells,^107,108^ that they display hemocompatibility,^109^ and that the quantity of copper released is considered safe for the human body.^87^

Additionally, CuS can form heterostructures to improve the properties of the composites, as reported by Park et al.^102^ This group combined CuS with AuNPs in a core–shell hybrid system with a dual plasmonic LSPR absorption band in the visible and NIR for photothermal purpose. Au@CuS NPs were blended in PDMS, resulting in a photothermal material able to inactivate *E. coli* under NIR irradiation after only 10 s. Wang et al.^105^ proposed a polyurethane (PU) film with CuS and Ti_3_C_2_T_*x*_ MXene heterojunctions included with photocatalytic antibacterial and photothermal properties. Moreover, the PU films have improved mechanical properties thanks to the coordination bonds with Cu^2+^ that also prevent the release of heavy metal ions. Despite the local increased temperature up to ≈120 °C in 600 s when irradiated in the NIR, the researchers did not perform any photothermal antibacterial evaluation. Another example of a nano-heterojunction reported in the literature is a combination of CuS with the photodynamic antibacterial agent graphitic carbon nitride (g-C_3_N_4_-PDA agent), included into poly-l-lactide (PLLA) scaffolds.^106^ CuS in this structure has multiple roles: photothermal antibacterial agent under NIR irradiation and inhibitor of electron–hole recombination of g-C_3_N_4_ for easier ROS generation.

Copper selenides belong to the family of copper chalcogenides with photothermal conversion efficiency properties and can be used for the same purpose. Wang et al.^99^ proposed a low cost green synthesis of an efficient photothermal antibacterial membrane by including bio-CuSe NPs in polyvinylidene fluoride (PVDF) substrate. The temperature of water in contact with these promising bio-CuSe/PVDF substrates increases by ≈15 °C when irradiated with a 1064 nm and 3 W/cm^2^ laser for 30 min. This slow increase has been demonstrated sufficient to exert an antibacterial effect against *S. aureus* and *B. subtilis*.

## Prussian Blue Nanomaterials

**Figure 4:**
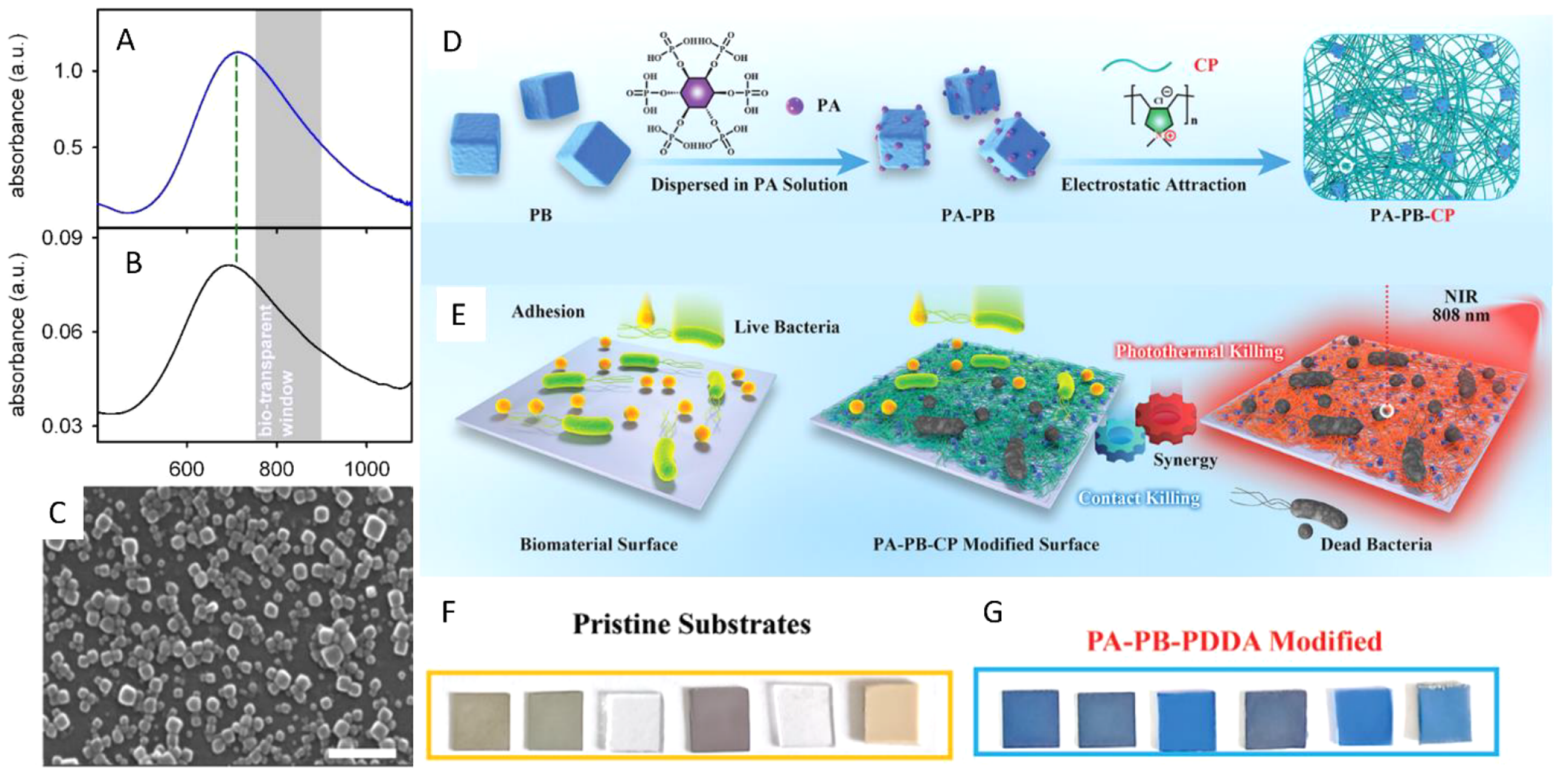
(A and B) Absorption spectra of (A) PBNPs colloidal solution and (B) dry (air interface) self-assembled monolayer on glass, prepared using PBNPs with the absorption spectrum. Adapted from ref (17). Creative Commons Attribution 4.0 https://creativecommons.org/licenses/by/4.0/ (accessed on 12 February 2024). (C) SEM micrographs of PBNPs grafted on glass (scale bar, 250 nm). Adapted from ref (74) with permission from Royal Society of Chemistry. (D) Preparation of PA–PB–CP network coating for antibacterial application. (E) Schematic illustration of the “contact-killing” and photothermal antibacterial ability of the PA–PB–CP network coating. (F and G) Pictures of Ti, SS, glass, Si, PDMS, and PEEK (from left to right) surfaces before and after deposition of the PA-PB-PDDA coating. Adapted from ref (114). Creative Commons Attribution 4.0 https://creativecommons.org/licenses/by/4.0/ (accessed on 12 February 2024).

Prussian blue (PB) nanomaterials have been used as inorganic photothermal agents (PTAs) in different applications. This coordination polymer has been studied since the 18th century and in 2003 was approved by the FDA for use in humans.^110^ PB can be easily prepared in colloidal form,^17,111,112^ showing a very strong and broad absorption band centered at ≈700 nm with a tail in the NIR region due to a charge transfer transition between Fe(II) and Fe(III) centers (Figure 4A and B). This charge transfer band allows for efficient absorption of light in the NIR, especially in the so-called “biotransparent window” (*i.e.* 750–900 nm).^17,30,112^ When PB is prepared in colloidal form, NIR light absorption leads to a local increase of temperature around Prussian blue NPs (PBNPs) due to thermal relaxation.^17,30,111,112^ The biocompatibility, nontoxicity, in vivo stability, and photothermal properties of PB nanomaterials make them very attractive for in vivo use in the field of photothermal bactericidal surfaces, by decorating biomedical devices, implants, and air purification systems with these PTAs. Table 4 summarizes several Prussian blue photothermal nanomaterials, specifying typical substrates, preparation methods, laser wavelengths used, and bacteria tested. The first study of Prussian blue as photothermal antibacterial surface was conducted in our laboratory by grafting Prussian blue nanoparticles on a glass surface via electrostatic assembly with a good coverage and homogeneous distribution of PBNPs (Figure 4C).^112^ The irradiation of these functionalized surfaces for 30 min with a laser centered at 808 nm and with power 0.25 W/cm^2^ (below ANSI limitation) allows reaching a Δ*T* sufficient to exert good photothermal properties against Gram– (*E. coli*) and Gram+ (*S. aureus*) bacteria. Likewise, we prepared a hybrid material containing both PBNPs and Ag^+^ ions with the same approach to improve antibacterial properties, resulting in a synergic effect that enhanced the photothermal microbicidal effect against Gram+ bacteria (although a very small quantity of loaded Ag^+^). To improve better the photothermal antibacterial effect in another recent work of our laboratory,^74^ we opted to functionalize glass by a layer-by-layer approach combining one layer of Prussian blue covered with a layer of silica (to protect PBNPs) and a layer of silver nanoparticles. The antibacterial effect is increased and accelerated in the presence of irradiation at 808 nm with respect to the ANSI limitation, and it is even higher than our past work. In both cases, PBNPs were synthesized before and then grafted on the surface with electrostatic self-assembly. Ngo et al.,^113^ instead, exploited a one-pot approach, resulting in the direct synthesis of PBNPs by the simultaneous addition of precursor on three different surfaces: bare gold, cysteamine-functionalized gold, and glass- supported lipid bilayers (SLBs). Despite the isolated formation of PB nanopyramids on the bare gold surfaces, they obtained optimal and regular coverage of cubic PBNPs shape controlled in the case of gold functionalized with cysteamine. Moreover, the goal was achieved also with the SLB surfaces by modulating the cationic lipid concentration of the surfaces by the presence of favorable electrostatic interactions between negatively charged PBNPs and positive lipids. As a matter of fact, they noticed that the Pb nanopyramid on bare gold did not have any photothermal effect. Concerning the self-assembled monolayer prepared by Dacarro et al.^112^ and Doveri et al.,^74^ the photothermal effect is Δ*T* = 13.3(9) at 808 nm and 0.26 W/cm^2^, while that of the surfaces with the one pot synthesis obtained by Ngo et al.^113^ is ≈10 K with the same conditions, meaning they obtained the same coverage. However, the last did not evaluate the photothermal bactericidal effect of their substrates. Of course, by increasing the irradiance of the laser and overcoming the ANSI limitation, Ngo et al. reached Δ*T* = 70 K at 1.5 W/cm^2^.^113^ PB photothermal surfaces can also be obtained by blending colloidal PBNPs in polymers like poly(vinyl alcohol) (PVA), resulting in bluish PVA-PB films obtained by a casting method with a uniform nanoparticles distribution and low degree of aggregation (concentration of PBNPs 3 mM). The local increase of these PVA-PB films is Δ*T* ≅ 78 °C in 10 s under the irradiation of a laser centered at 700 nm with *I* = 0.3W/cm^2^ that allowed exertion of an efficient antibacterial effect on *Pseudomonas aeruginosa*.^115^ Wang et al.^116^ blended sodium nitroprusside doped mesoporous Prussian blue nanoparticles (SNP-PBNPs) in chitosan/PVA to obtain electrospun nanofiber for wound healing and antibacterial application. The scaffold with 2 mg/mL of SNP-PBNPs irradiated at 808 nm with an irradiance of 0.5 W/cm^2^ reached a Δ*T* ≅ 50 K, and their inhibition rate of bacteria when NIR treated reached 88.4% and 85.9% for *S. aureus* and *E. coli*, respectively. They suppose that the antibacterial efficiency is due to a synergistic effect between PTT and increased NO release after irradiation. However, these films have the limitations of not being able to adhere to nonplanar surfaces and require large amounts of nanoparticles, and the solution found by Borzenkov et al.^46^ was to cover glass disks by the spray coating technique. The local increase of these homogeneous glasses irradiated with a laser at 700 nm with *I* = 0.35 W/cm^2^ was <70 °C, sufficient to photothermally eradicate *P. aeruginosa* and to have a remarkable effect on *S. aureus*. Comparing PBNPs sprayed glasses with glasses functionalized with gold nanostars prepared in the same way, it was found that the PB ones are more efficient.^46^ Recently He et al.^114^ functionalized PBNPs with phytic acid (PA) by metal chelation and cationic polymers (CPs) through electrostatic interaction to form a network able to coat different surfaces (Figure 4D), such as titanium (Ti), stainless steel (SS), glass, silicone (Si), polydimethylsiloxane (PDMS), and poly(ether ether ketone) (PEEK), and different shapes, by surface adherence and gravitational deposition as depicted in Figure 4F and G. Figure 4E shows a schematic illustration of the possible dynamic and photothermal antibacterial capability of the PA–PB–CP network coating. The photothermal effect of resistant surfaces coated with the PA–PB–CP network was assayed by irradiation with a laser at 808 nm and 0.75 W/cm^2^ irradiation, resulting that the best surfaces achieved Δ*T*= 54.1 K after 5 min of irradiation. Moreover, hyperthermal action combined with contact effects enabled fast eradication of *E. coli* and *S. aureus* upon irradiation with NIR light, although the irradiance levels needed to exceed the ANSI limitation for skin exposure.

**Table 4:** Summary of Photothermal Antibacterial Surfaces Based on Prussian Blue Nanomaterials

PTA	Substrate	Functionalization strategy	Antibacterial mechanism	Irradiation	Bacteria	Ref
PBNPs and PBNPs+Ag^+^	glass	self-assembly	PTTPTT+Ag^+^	808 nm, 0.25 W/cm^2^	*E. coli*, *S. aureus*	(112)
PBNPs	PVA	blending	PTT	700 nm, 0.3 W/cm^2^	*P. aeruginosa*	(115)
PBNPs	glass	spray coating	PTT	700 nm, 0.35 W/cm^2^	*P. aeruginosa*, *S. aureus*	(46)
PBNPs	Au, functionalized Au and SLB on glass	self-assembly	PTT	808 nm, 0.27–1.50 W/cm^2^		(113)
SNP-PBNPs	CSPVA scaffolds	blending	PTT+NO	808 nm, 1.0 W/cm^2^	*E. coli*, *S. aureus*	(116)
PBNPs+AgNPs	glass	self-assembly	PTT+Ag^+^	808 nm, 0.26 W/cm^2^	*E. coli*, *S. aureus*	(74)
PBNPs	Ti, steel, glass, Si, PDMS, PEEK	self-assembly	PTT	808 nm, 0.75 W/cm^2^	*E. coli*, *S. aureus*	(114)

## Carbon-Based Materials

Carbon-based nanomaterials (CBNs) are constantly increasing their popularity in several fields, due to the high availability and low cost of carbon, its inertness, and the interesting properties that its nanomaterials and allotropic forms show. The first CBNs reported to have an antibacterial effect are single-^117^ and multiwalled^118^ carbon nanotubes (SWCNTs-MWCNTs),^119^ fullerenes (C60),^120^ and graphene/graphite-based materials (graphite, graphite oxide, graphene oxide, and reduced graphene oxide).^121^ The activity of these materials is mainly related to CBN–membrane interactions, with CBNs being capable of penetrating and rupturing the membranes and walls of bacterial cells.^122^ Other mechanisms involved in the antimicrobial activity of CBNs are oxidative stress, inhibition of metabolism, and photocatalytic effect. A complete discussion on these mechanisms of action can be found in a comprehensive review from Gong and co-workers.^123^ Focusing on the photothermal properties, CNTs in general have broad absorption bands in the visible and NIR regions of the spectrum. Among the different types of CNTs, graphitic materials typically show the highest light-to-heat conversion (compared to diamond-like materials like nanodiamonds).^124^ It is also important to stress that in CNTs, a pure photothermal effect is often coupled with photodynamic properties and ROS generation. The two different contributions to the antimicrobial effect are often difficult to separate and are usually synergic.

CNTs can be used as such in colloidal dispersion, or they can be easily combined to other materials in composites or grafted on materials for surface functionalization. CNTs are easily coupled to a great variety of materials. In many cases, the preparation of a composite material does not need specific and complex synthetic pathways.

Suo et al.,^125^ for example, prepared dentin-nanotubes composites just by soaking human dentin samples in SWNTs and MWNTs water suspensions. SWNTs coated dentin showed good prevention of *S. mutans* adhesion. Similarly, Musico et al.^126^ included graphene and graphene oxide in membrane filters. The authors report good antibacterial activity against *E. coli* and *B. subtilis*. The activity is enhanced by the presence of poly(*N*-vinylcarbazole) (PVK) and is related to generation of ROS.

To our knowledge, in the literature only a few examples of photothermal antibacterial surfaces based on carbon nanomaterials have been reported so far. Two interesting examples were recently published by Lin and co-workers.^127,128^ In these papers, densely packed carbon-black particles were deposited from candle soot on a glass slide. The carbon layer was made more stable with a silica overlayer and further coated with a fluorinated polymer to impart superhydrophobicity to the surface. This rather simple preparation led to photothermally active samples capable of a temperature increase of 50 °C when irradiated at 808 nm with a 1.5 W/cm^2^ irradiance for 10 min. The surfaces lead to complete eradication of *E. coli* and *S. aureus* strains under NIR irradiation, combined with the possibility of releasing dead bacterial cells thanks to the presence of antifouling polymers on the surface.

Another interesting example of a CNT-based photothermal surface was published by Zhang^129^ and co-workers. The authors prepared a red phosphorus/graphene hybrid nanomaterial on titanium surfaces. Irregular pyramids of crystalline P on Ti were prepared by chemical vapor deposition (CVD) and coated in shellac that is transitioned into GO. Prepared substrates showed photothermal, photocatalytic, and photoelectrochemical activity. When irradiated with simulated sunlight (SSL) for 20 min, the substrates showed an inactivation of about 99.9% of *S. aureus* and *E. coli* strains. Antibacterial activity tests were also conducted at different irradiation wavelengths (SSL was filtered with >410 nm and >800 nm long pass filters). Good effects were still present also when UV and visible light are filtered. As we previously mentioned, though, the authors could not exactly determine if the effects were due mainly to hyperthermia, generation of ROS, or a combination/synergy of the two.

## Conclusions

In this review, we discuss the state of the art of photothermally active nanostructured surfaces. The field is rather new, but the materials used could soon find applications in several fields, including permanent or indwelling medical devices (*e.g*. prostheses, catheters, implants), every-day household surfaces, and industrial settings. Each field of application obviously has different requirements in terms of chemical and mechanical stability, composition of the material, and condition of irradiation. Considerable effort has been devoted to the development of systems based on noble-metal plasmonic NPs, showing promising results; however, the economic cost of these critical raw materials may become prohibitive for scale-up applications. Alternative materials such as carbon-based or Prussian blue NPs are gaining attention due to their enhanced biocompatibility and lower cost, but several issues related to the chemical and physical stability of these systems prevent versatile uses for real-life settings. Several applications in biomedical or household environments will require a more accurate investigation of nanoparticle leaching and nanotoxicity to overcome strong regulatory barriers. The need for intense light sources to activate photothermal responses may also represent a limitation for cost-effective applications that may find their way through innovation and industrial development. Even though most of the studies reported here are proof of principle studies at very low TRL, they pave the way to more advanced and widespread applications, and we believe that this new class of materials, able to exert switchable effects activated by harmless light sources, can offer a valid alternative to traditional methods for fighting critical issues such as microbial infections that spread from surfaces or to improve light-to-heat conversion in building materials for a more sustainable future.

## Abbreviations

AMR: anti-microbial resistance
ANSI: American National Standards Institute
GNS: gold nanostars
ICNIRP: International Commission On Non Ionizing Radiation Protection
LSPR: localized surface plasmon resonance
MES: 3-(trimethoxysilyl)propyl methacrylate
NIR: near-infrared
PAM-*co*-PAOSA: polyacrylamide-*co*-poly(acrylic anhydride-modified oxidized sodium alginate)
PAN: polyacrylonitrile
PDMS: polydimethylsiloxane
PEEK: poly(ether ether ketone)
pNIPAM: poly(*N*-isopropylacryl-amide)
PTA: photothermal agent
PTD: photodynamic therapy
PTT: photothermal therapy
PU: polyurethane
PVA: poly(vinyl alcohol)
PVDF: poly-vinylidene fluoride
TRIM: thermal-disrupting interface induced mitigation
WHO: World Health Organization

## References

[ref1] Silver S.; Phung L. T.; Silver G. Silver as Biocides in Burn and Wound Dressings and Bacterial Resistance to Silver Compounds. J. Ind. Microbiol. Biotechnol. 2006, 33 (7), 627–634. 10.1007/s10295-006-0139-7. 16761169

[ref2] Barillo D. J.; Marx D. E. Silver in Medicine: A Brief History BC 335 to Present. Burns 2014, 40, S3–S8. 10.1016/j.burns.2014.09.009. 25418435

[ref3] Antimicrobial resistance. https://www.who.int/news-room/fact-sheets/detail/antimicrobial-resistance (accessed 2024-01-10).

[ref4] Murray C. J. L.; Ikuta K. S.; Sharara F.; Swetschinski L.; Robles Aguilar G.; Gray A.; Han C.; Bisignano C.; Rao P.; Wool E.; Johnson S. C.; Browne A. J.; Chipeta M. G.; Fell F.; Hackett S.; Haines-Woodhouse G.; Kashef Hamadani B. H.; Kumaran E. A. P.; McManigal B.; Achalapong S.; Agarwal R.; Akech S.; Albertson S.; Amuasi J.; Andrews J.; Aravkin A.; Ashley E.; Babin F.-X.; Bailey F.; Baker S.; Basnyat B.; Bekker A.; Bender R.; Berkley J. A.; Bethou A.; Bielicki J.; Boonkasidecha S.; Bukosia J.; Carvalheiro C.; Castañeda-Orjuela C.; Chansamouth V.; Chaurasia S.; Chiurchiù S.; Chowdhury F.; Clotaire Donatien R.; Cook A. J.; Cooper B.; Cressey T. R.; Criollo-Mora E.; Cunningham M.; Darboe S.; Day N. P. J.; De Luca M.; Dokova K.; Dramowski A.; Dunachie S. J.; Duong Bich T.; Eckmanns T.; Eibach D.; Emami A.; Feasey N.; Fisher-Pearson N.; Forrest K.; Garcia C.; Garrett D.; Gastmeier P.; Giref A. Z.; Greer R. C.; Gupta V.; Haller S.; Haselbeck A.; Hay S. I.; Holm M.; Hopkins S.; Hsia Y.; Iregbu K. C.; Jacobs J.; Jarovsky D.; Javanmardi F.; Jenney A. W. J.; Khorana M.; Khusuwan S.; Kissoon N.; Kobeissi E.; Kostyanev T.; Krapp F.; Krumkamp R.; Kumar A.; Kyu H. H.; Lim C.; Lim K.; Limmathurotsakul D.; Loftus M. J.; Lunn M.; Ma J.; Manoharan A.; Marks F.; May J.; Mayxay M.; Mturi N.; Munera-Huertas T.; Musicha P.; Musila L. A.; Mussi-Pinhata M. M.; Naidu R. N.; Nakamura T.; Nanavati R.; Nangia S.; Newton P.; Ngoun C.; Novotney A.; Nwakanma D.; Obiero C. W.; Ochoa T. J.; Olivas-Martinez A.; Olliaro P.; Ooko E.; Ortiz-Brizuela E.; Ounchanum P.; Pak G. D.; Paredes J. L.; Peleg A. Y.; Perrone C.; Phe T.; Phommasone K.; Plakkal N.; Ponce-de-Leon A.; Raad M.; Ramdin T.; Rattanavong S.; Riddell A.; Roberts T.; Robotham J. V.; Roca A.; Rosenthal V. D.; Rudd K. E.; Russell N.; Sader H. S.; Saengchan W.; Schnall J.; Scott J. A. G.; Seekaew S.; Sharland M.; Shivamallappa M.; Sifuentes-Osornio J.; Simpson A. J.; Steenkeste N.; Stewardson A. J.; Stoeva T.; Tasak N.; Thaiprakong A.; Thwaites G.; Tigoi C.; Turner C.; Turner P.; van Doorn H. R.; Velaphi S.; Vongpradith A.; Vongsouvath M.; Vu H.; Walsh T.; Walson J. L.; Waner S.; Wangrangsimakul T.; Wannapinij P.; Wozniak T.; Young Sharma T. E. M. W.; Yu K. C.; Zheng P.; Sartorius B.; Lopez A. D.; Stergachis A.; Moore C.; Dolecek C.; Naghavi M. Global Burden of Bacterial Antimicrobial Resistance in 2019: A Systematic Analysis. Lancet 2022, 399 (10325), 629–655. 10.1016/S0140-6736(21)02724-0. 35065702 PMC8841637

[ref5] The true death toll of COVID-19. https://www.who.int/data/stories/the-true-death-toll-of-covid-19-estimating-global-excess-mortality (accessed 2024-01-11).

[ref6] Lei H.; Li Y.; Xiao S.; Yang X.; Lin C.; Norris S. L.; Wei D.; Hu Z.; Ji S. Logistic Growth of a Surface Contamination Network and Its Role in Disease Spread. Sci. Rep. 2017, 7 (1), 14826 10.1038/s41598-017-13840-z. 29093534 PMC5665872

[ref7] Haque M.; Sartelli M.; McKimm J.; Bakar M. A. Health Care-Associated Infections - An Overview. Infect. Drug Resist. 2018, 11, 2321–2333. 10.2147/IDR.S177247. 30532565 PMC6245375

[ref8] Health Care-Associated Infections. https://www.hhs.gov/oidp/topics/health-care-associated-infections/index.html (accessed 2024-01-11).

[ref9] World Health Organization. Report on the Burden of Endemic Health Care-Associated Infection Worldwide: Clean Care Is Safer Care. World Heal. Organ. 2011, 3, 1–34.

[ref10] Cobrado L.; Silva-Dias A.; Azevedo M. M.; Rodrigues A. G. High-Touch Surfaces: Microbial Neighbours at Hand. Eur. J. Clin. Microbiol. Infect. Dis. 2017, 36 (11), 2053–2062. 10.1007/s10096-017-3042-4. 28647859 PMC7087772

[ref11] Reduce risk from surfaces. CDC Website. https://www.cdc.gov/hai/prevent/environment/surfaces.html (accessed 2024-01-16).

[ref12] Hao S.; Han H.; Yang Z.; Chen M.; Jiang Y.; Lu G.; Dong L.; Wen H.; Li H.; Liu J.; Wu L.; Wang Z.; Wang F. Recent Advancements on Photothermal Conversion and Antibacterial Applications over MXenes-Based Materials. Nano-Micro Lett. 2022, 14 (1), 178 10.1007/s40820-022-00901-w. PMC9402885 36001173

[ref13] Zou Y.; Zhang Y.; Yu Q.; Chen H. Photothermal Bactericidal Surfaces: Killing Bacteria Using Light Instead of Biocides. Biomater. Sci. 2021, 9 (1), 10–22. 10.1039/D0BM00617C. 32525140

[ref14] Katzenberger R. H.; Rösel A.; Vonberg R.-P. Bacterial Survival on Inanimate Surfaces: A Field Study. BMC Res. Notes 2021, 14 (1), 97 10.1186/s13104-021-05492-0. 33722269 PMC7962391

[ref15] Cui X.; Ruan Q.; Zhuo X.; Xia X.; Hu J.; Fu R.; Li Y.; Wang J.; Xu H. Photothermal Nanomaterials: A Powerful Light-to-Heat Converter. Chem. Rev. 2023, 123 (11), 6891–6952. 10.1021/acs.chemrev.3c00159. 37133878 PMC10273250

[ref16] Pallavicini P.; Chirico G.; Taglietti A. Harvesting Light To Produce Heat: Photothermal Nanoparticles for Technological Applications and Biomedical Devices. Chem. - A Eur. J. 2021, 27 (62), 15361–15374. 10.1002/chem.202102123. PMC8597085 34406677

[ref17] Dacarro G.; Taglietti A.; Pallavicini P. Prussian Blue Nanoparticles as a Versatile Photothermal Tool. Molecules 2018, 23 (6), 1414 10.3390/molecules23061414. 29891819 PMC6099709

[ref18] Yun B.; Zhu H.; Yuan J.; Sun Q.; Li Z. Synthesis, Modification and Bioapplications of Nanoscale Copper Chalcogenides. J. Mater. Chem. B 2020, 8 (22), 4778–4812. 10.1039/D0TB00182A. 32226981

[ref19] Hemmer E.; Benayas A.; Légaré F.; Vetrone F. Exploiting the Biological Windows: Current Perspectives on Fluorescent Bioprobes Emitting above 1000 Nm. Nanoscale Horizons 2016, 1 (3), 168–184. 10.1039/C5NH00073D. 32260620

[ref20] Prodi L.; Rampazzo E.; Rastrelli F.; Speghini A.; Zaccheroni N. Imaging Agents Based on Lanthanide Doped Nanoparticles. Chem. Soc. Rev. 2015, 44 (14), 4922–4952. 10.1039/C4CS00394B. 26090530

[ref21] American National Standard for Safe Use of Lasers (ANSI 136.1). The Laser Institute of America 2000.

[ref22] Ziegelberger G. ICNIRP Guidelines on Limits of Exposure to Laser Radiation of Wavelengths between 180 Nm and 1,000 Μm. Health Phys. 2013, 105 (3), 271–295. 10.1097/HP.0b013e3182983fd4. 30522251

[ref23] Xu J.-W.; Yao K.; Xu Z.-K. Nanomaterials with a Photothermal Effect for Antibacterial Activities: An Overview. Nanoscale 2019, 11 (18), 8680–8691. 10.1039/C9NR01833F. 31012895

[ref24] Bai X.; Yang Y.; Zheng W.; Huang Y.; Xu F.; Bao Z. Synergistic Photothermal Antibacterial Therapy Enabled by Multifunctional Nanomaterials: Progress and Perspectives. Mater. Chem. Front. 2023, 7 (3), 355–380. 10.1039/D2QM01141G.

[ref25] Xu J. W.; Yao K.; Xu Z. K. Nanomaterials with a Photothermal Effect for Antibacterial Activities: An Overview. Nanoscale 2019, 11 (18), 8680–8691. 10.1039/C9NR01833F. 31012895

[ref26] Rusu A.; Munteanu A.-C.; Arbǎnaşi E.-M.; Uivarosi V. Overview of Side-Effects of Antibacterial Fluoroquinolones: New Drugs versus Old Drugs, a Step Forward in the Safety Profile?. Pharmaceutics 2023, 15 (3), 804 10.3390/pharmaceutics15030804. 36986665 PMC10056716

[ref27] Yang Q.; Gao Y.; Ke J.; Show P. L.; Ge Y.; Liu Y.; Guo R.; Chen J. Antibiotics: An Overview on the Environmental Occurrence, Toxicity, Degradation, and Removal Methods. Bioengineered 2021, 12 (1), 7376–7416. 10.1080/21655979.2021.1974657. 34612807 PMC8806427

[ref28] Dahiya D.; Nigam P. S. Antibiotic-Therapy-Induced Gut Dysbiosis Affecting Gut Microbiota—Brain Axis and Cognition: Restoration by Intake of Probiotics and Synbiotics. Int. J. Mol. Sci. 2023, 24 (4), 3074 10.3390/ijms24043074. 36834485 PMC9959899

[ref29] Liu W.; Yu Y.; Cheng W.; Zhou M.; Cui L.; Wang P.; Wang Q. Melanin-like Nanoparticles Loaded with Ag NPs for Rapid Photothermal Sterilization and Daily Protection of Textiles. Colloids Surfaces B Biointerfaces 2022, 219, 112829 10.1016/j.colsurfb.2022.112829. 36137339

[ref30] Borzenkov M.; Pallavicini P.; Chirico G. Photothermally Active Inorganic Nanoparticles: From Colloidal Solutions to Photothermally Active Printed Surfaces and Polymeric Nanocomposite Materials. Eur. J. Inorg. Chem. 2019, 2019 (41), 4397–4404. 10.1002/ejic.201900836.

[ref31] Kohout C.; Santi C.; Polito L. Anisotropic Gold Nanoparticles in Biomedical Applications. Int. J. Mol. Sci. 2018, 19 (11), 3385 10.3390/ijms19113385. 30380664 PMC6274885

[ref32] Hang Y.; Wang A.; Wu N. Plasmonic Silver and Gold Nanoparticles: Shape- and Structure-Modulated Plasmonic Functionality for Point-of-Caring Sensing, Bio-Imaging and Medical Therapy. Chem. Soc. Rev. 2024, 53, 2932–2971. 10.1039/D3CS00793F. 38380656 PMC11849058

[ref33] Yusufbeyoǧlu S.; Cinar V.; Ildiz N.; Hamurcu Z.; Ocsoy İ.; Kilic A. B. The Use of Conjugated Gold Nanorods with Reduced Toxicity in Photothermal Therapy for MRSA. ChemistrySelect 2024, 9 ( (11), ), e202304893 10.1002/slct.202304893.

[ref34] Huang X.; Jain P. K.; El-Sayed I. H.; El-Sayed M. A. Plasmonic Photothermal Therapy (PPTT) Using Gold Nanoparticles. Lasers Med. Sci. 2008, 23 (3), 217–228. 10.1007/s10103-007-0470-x. 17674122

[ref35] Demishkevich E.; Zyubin A.; Seteikin A.; Samusev I.; Park I.; Hwangbo C. K.; Choi E. H.; Lee G. J. Synthesis Methods and Optical Sensing Applications of Plasmonic Metal Nanoparticles Made from Rhodium, Platinum, Gold, or Silver. Materials (Basel). 2023, 16 (9), 3342 10.3390/ma16093342. 37176223 PMC10180225

[ref36] He W.; Huang X.; Ma X.; Zhang J. Significant Temperature Effect on the LSPR Properties of Noble Metal Nanoparticles. J. Opt. 2022, 51 (1), 142–153. 10.1007/s12596-021-00766-z.

[ref37] Scarabelli L.; Grzelczak M.; Liz-Marzán L. M. Tuning Gold Nanorod Synthesis through Prereduction with Salicylic Acid. Chem. Mater. 2013, 25 (21), 4232–4238. 10.1021/cm402177b.

[ref38] Scarabelli L.; Sánchez-Iglesias A.; Pérez-Juste J.; Liz-Marzán L. M. A ‘Tips and Tricks’ Practical Guide to the Synthesis of Gold Nanorods. J. Phys. Chem. Lett. 2015, 6 (21), 4270–4279. 10.1021/acs.jpclett.5b02123. 26538043

[ref39] Pallavicini P.; Donà A.; Casu A.; Chirico G.; Collini M.; Dacarro G.; Falqui A.; Milanese C.; Sironi L.; Taglietti A. Triton X-100 for Three-Plasmon Gold Nanostars with Two Photothermally Active NIR (near IR) and SWIR (Short-Wavelength IR) Channels. Chem. Commun. 2013, 49 (56), 6265 10.1039/c3cc42999g. 23728398

[ref40] Atta S.; Beetz M.; Fabris L. Understanding the Role of AgNO 3 Concentration and Seed Morphology in the Achievement of Tunable Shape Control in Gold Nanostars. Nanoscale 2019, 11 (6), 2946–2958. 10.1039/C8NR07615D. 30693922

[ref41] Ortiz-Castillo J. E.; Gallo-Villanueva R. C.; Madou M. J.; Perez-Gonzalez V. H. Anisotropic Gold Nanoparticles: A Survey of Recent Synthetic Methodologies. Coord. Chem. Rev. 2020, 425, 213489 10.1016/j.ccr.2020.213489.

[ref42] Casu A.; Cabrini E.; Donà A.; Falqui A.; Diaz-Fernandez Y.; Milanese C.; Taglietti A.; Pallavicini P. Controlled Synthesis of Gold Nanostars by Using a Zwitterionic Surfactant. Chem. - A Eur. J. 2012, 18 (30), 9381–9390. 10.1002/chem.201201024. 22736477

[ref43] Toci G.; Olgiati F.; Pallavicini P.; Diaz Fernandez Y. A.; De Vita L.; Dacarro G.; Grisoli P.; Taglietti A. Gold Nanostars Embedded in PDMS Films: A Photothermal Material for Antibacterial Applications. Nanomaterials 2021, 11 (12), 3252 10.3390/nano11123252. 34947603 PMC8707359

[ref44] Pallavicini P.; Donà A.; Taglietti A.; Minzioni P.; Patrini M.; Dacarro G.; Chirico G.; Sironi L.; Bloise N.; Visai L.; Scarabelli L. Self-Assembled Monolayers of Gold Nanostars: A Convenient Tool for near-IR Photothermal Biofilm Eradication. Chem. Commun. 2014, 50 (16), 1969–1971. 10.1039/C3CC48667B. 24406855

[ref45] Rovati D.; Albini B.; Galinetto P.; Grisoli P.; Bassi B.; Pallavicini P.; Dacarro G.; Taglietti A. High Stability Thiol-Coated Gold Nanostars Monolayers with Photo-Thermal Antibacterial Activity and Wettability Control. Nanomaterials 2019, 9 (9), 1288 10.3390/nano9091288. 31505833 PMC6781089

[ref46] Borzenkov M.; Chirico G.; Pallavicini P.; Sperandeo P.; Polissi A.; Dacarro G.; Doveri L.; Collini M.; Sironi L.; Bouzin M.; D’Alfonso L. Nanocomposite Sprayed Films with Photo-Thermal Properties for Remote Bacteria Eradication. Nanomaterials 2020, 10 (4), 786 10.3390/nano10040786. 32325935 PMC7221876

[ref47] Borzenkov M.; Moros M.; Tortiglione C.; Bertoldi S.; Contessi N.; Faré S.; Taglietti A.; D’Agostino A.; Pallavicini P.; Collini M.; Chirico G. Fabrication of Photothermally Active Poly(Vinyl Alcohol) Films with Gold Nanostars for Antibacterial Applications. Beilstein J. Nanotechnol. 2018, 9 (1), 2040–2048. 10.3762/bjnano.9.193. 30116694 PMC6071708

[ref48] Pallavicini P.; Bassi B.; Chirico G.; Collini M.; Dacarro G.; Fratini E.; Grisoli P.; Patrini M.; Sironi L.; Taglietti A.; Moritz M.; Sorzabal-Bellido I.; Susarrey-Arce A.; Latter E.; Beckett A. J.; Prior I. A.; Raval R.; Diaz Fernandez Y. A. Modular Approach for Bimodal Antibacterial Surfaces Combining Photo-Switchable Activity and Sustained Biocidal Release. Sci. Rep. 2017, 7 (1), 5259 10.1038/s41598-017-05693-3. 28701753 PMC5507905

[ref49] Yang T.; Wang D.; Liu X. Assembled Gold Nanorods for the Photothermal Killing of Bacteria. Colloids Surfaces B Biointerfaces 2019, 173, 833–841. 10.1016/j.colsurfb.2018.10.060. 30551299

[ref50] Grisoli P.; De Vita L.; Milanese C.; Taglietti A.; Diaz Fernandez Y.; Bouzin M.; D’Alfonso L.; Sironi L.; Rossi S.; Vigani B.; Sperandeo P.; Polissi A.; Pallavicini P. PVA Films with Mixed Silver Nanoparticles and Gold Nanostars for Intrinsic and Photothermal Antibacterial Action. Nanomaterials 2021, 11 (6), 1387 10.3390/nano11061387. 34070273 PMC8225135

[ref51] Hu B.; Berkey C.; Feliciano T.; Chen X.; Li Z.; Chen C.; Amini S.; Nai M. H.; Lei Q.; Ni R.; Wang J.; Leow W. R.; Pan S.; Li Y.; Cai P.; Miserez A.; Li S.; Lim C. T.; Wu Y.; Odom T. W.; Dauskardt R. H.; Chen X. Thermal Disrupting Interface Mitigates Intercellular Cohesion Loss for Accurate Topical Antibacterial Therapy. Adv. Mater. 2020, 32 (12), 1–10. 10.1002/adma.201907030. PMC7702719 32072703

[ref52] Khantamat O.; Li C. H.; Yu F.; Jamison A. C.; Shih W. C.; Cai C.; Lee T. R. Gold Nanoshell-Decorated Silicone Surfaces for the near-Infrared (NIR) Photothermal Destruction of the Pathogenic Bacterium E. Faecalis. ACS Appl. Mater. Interfaces 2015, 7 (7), 3981–3993. 10.1021/am506516r. 25611157

[ref53] Qu Y.; Wei T.; Zhao J.; Jiang S.; Yang P.; Yu Q.; Chen H. Regenerable Smart Antibacterial Surfaces: Full Removal of Killed Bacteria via a Sequential Degradable Layer. J. Mater. Chem. B 2018, 6 (23), 3946–3955. 10.1039/C8TB01122B. 32254323

[ref54] Zheng L.; Gu B.; Li S.; Luo B.; Wen Y.; Chen M.; Li X.; Zha Z.; Zhang H.-T.; Wang X. An Antibacterial Hemostatic AuNPs@corn Stalk/Chitin Composite Sponge with Shape Recovery for Promoting Wound Healing. Carbohydr. Polym. 2022, 296, 119924 10.1016/j.carbpol.2022.119924. 36088022

[ref55] Zhao P.; Feng Y.; Zhou Y.; Tan C.; Liu M. Gold@Halloysite Nanotubes-Chitin Composite Hydrogel with Antibacterial and Hemostatic Activity for Wound Healing. Bioact. Mater. 2023, 20, 355–367. 10.1016/j.bioactmat.2022.05.035. 35784635 PMC9207301

[ref56] Bermúdez-Jiménez C.; Niño Martínez N.; Patiño Marín N.; Martínez Gutiérrez F.; Ruiz F.; Bach H.; Martínez Castañón G. Effective Control of Biofilms by Photothermal Therapy Using a Gold Nanorod Hydrogel. J. Biomed. Mater. Res. Part B Appl. Biomater. 2020, 108 (2), 333–342. 10.1002/jbm.b.34392. 31041849

[ref57] Zhao Y.-Q.; Sun Y.; Zhang Y.; Ding X.; Zhao N.; Yu B.; Zhao H.; Duan S.; Xu F.-J. Well-Defined Gold Nanorod/Polymer Hybrid Coating with Inherent Antifouling and Photothermal Bactericidal Properties for Treating an Infected Hernia. ACS Nano 2020, 14 (2), 2265–2275. 10.1021/acsnano.9b09282. 32017535

[ref58] Zhao Y.-Q.; Xiu Z.; Wu R.; Zhang L.; Ding X.; Zhao N.; Duan S.; Xu F.-J. A Near Infrared Responsive Quaternary Ammonium/Gold Nanorod Hybrid Coating with Enhanced Antibacterial Properties. Adv. NanoBiomed Res. 2022, 2 (11), 2200111 10.1002/anbr.202200111.

[ref59] Yang T.; Wang D.; Liu X. Antibacterial Activity of an NIR-Induced Zn Ion Release Film. J. Mater. Chem. B 2020, 8 (3), 406–415. 10.1039/C9TB02258A. 31850453

[ref60] Li L.; Li G.; Wu Y.; Lin Y.; Qu Y.; Wu Y.; Lu K.; Zou Y.; Chen H.; Yu Q.; Zhang Y. Dual-Functional Bacterial Cellulose Modified with Phase-Transitioned Proteins and Gold Nanorods Combining Antifouling and Photothermal Bactericidal Properties. J. Mater. Sci. Technol. 2022, 110, 14–23. 10.1016/j.jmst.2021.10.011.

[ref61] Haghighat Bayan M. A.; Rinoldi C.; Rybak D.; Zargarian S. S.; Zakrzewska A.; Cegielska O.; Põhako-Palu K.; Zhang S.; Stobnicka-Kupiec A.; Górny R. L.; Nakielski P.; Kogermann K.; De Sio L.; Ding B.; Pierini F. Engineering Surgical Face Masks with Photothermal and Photodynamic Plasmonic Nanostructures for Enhancing Filtration and On-Demand Pathogen Eradication. Biomater. Sci.. 2024, 12, 949 10.1039/D3BM01125A. 38221844

[ref62] D’Agostino A.; Taglietti A.; Grisoli P.; Dacarro G.; Cucca L.; Patrini M.; Pallavicini P. Seed Mediated Growth of Silver Nanoplates on Glass: Exploiting the Bimodal Antibacterial Effect by near IR Photo-Thermal Action and Ag+ Release. RSC Adv. 2016, 6 (74), 70414–70423. 10.1039/C6RA11608F.

[ref63] D’Agostino A.; Taglietti A.; Desando R.; Bini M.; Patrini M.; Dacarro G.; Cucca L.; Pallavicini P.; Grisoli P.; D’agostino A.; Taglietti A.; Desando R.; Bini M.; Patrini M.; Dacarro G.; Cucca L.; Pallavicini P.; Grisoli P. Bulk Surfaces Coated with Triangular Silver Nanoplates: Antibacterial Action Based on Silver Release and Photo-Thermal Effect. Nanomaterials 2017, 7 (1), 7 10.3390/nano7010007. 28336841 PMC5295197

[ref64] Liu Y.; Li F.; Guo Z.; Xiao Y.; Zhang Y.; Sun X.; Zhe T.; Cao Y.; Wang L.; Lu Q.; Wang J. Silver Nanoparticle-Embedded Hydrogel as a Photothermal Platform for Combating Bacterial Infections. Chem. Eng. J. 2020, 382, 122990 10.1016/j.cej.2019.122990.

[ref65] Li Y.; Yan Y.; Wang J.; Li L.; Tang F. Preparation of Silver Nanoparticles Decorated Mesoporous Silica Nanorods with Photothermal Antibacterial Property. Colloids Surfaces A Physicochem. Eng. Asp. 2022, 648 (May), 129242 10.1016/j.colsurfa.2022.129242.

[ref66] Merkl P.; Zhou S.; Zaganiaris A.; Shahata M.; Eleftheraki A.; Thersleff T.; Sotiriou G. A. Plasmonic Coupling in Silver Nanoparticle Aggregates and Their Polymer Composite Films for near -Infrared Photothermal Biofilm Eradication. ACS Appl. Nano Mater. 2021, 4 (5), 5330–5339. 10.1021/acsanm.1c00668. 34085032 PMC8165696

[ref67] Xu J.; Zhao Y.; Chen Y.; Chen Y.; Xie Z. H.; Munroe P. R. A Superhydrophilic, Light/Microwave-Absorbing Coating with Remarkable Antibacterial Efficacy. ACS Appl. Mater. Interfaces 2022, 14 (37), 42468–42482. 10.1021/acsami.2c11642. 36070517

[ref68] Liu H.; Xing F.; Zhou Y.; Yu P.; Xu J.; Luo R.; Xiang Z.; Maria Rommens P.; Liu M.; Ritz U. Nanomaterials-Based Photothermal Therapies for Antibacterial Applications. Mater. Des. 2023, 233, 112231 10.1016/j.matdes.2023.112231.

[ref69] Chen Y.; Hao X.; Lu Z.; Wang D. Near-IR-Regulated Composite Hydrogel with Real-Time Infection Monitoring and a Combined Antibacterial Effect for Efficient Wound Management. ACS Appl. Mater. Interfaces 2023, 15 (34), 40255–40266. 10.1021/acsami.3c08259. 37584530

[ref70] Talarska P.; Boruczkowski M.; Żurawski J. Current Knowledge of Silver and Gold Nanoparticles in Laboratory Research—Application, Toxicity, Cellular Uptake. Nanomaterials 2021, 11 ( (9), ), 2454 10.3390/nano11092454. 34578770 PMC8466515

[ref71] Sani A.; Cao C.; Cui D. Toxicity of Gold Nanoparticles (AuNPs): A Review. Biochem. Biophys. Reports 2021, 26, 100991 10.1016/j.bbrep.2021.100991. PMC8063742 33912692

[ref72] Murphy C. J.; Gole A. M.; Stone J. W.; Sisco P. N.; Alkilany A. M.; Goldsmith E. C.; Baxter S. C. Gold Nanoparticles in Biology: Beyond Toxicity to Cellular Imaging. Acc. Chem. Res. 2008, 41 (12), 1721–1730. 10.1021/ar800035u. 18712884

[ref73] Pallavicini P.; Dacarro G.; Taglietti A. Self-Assembled Monolayers of Silver Nanoparticles: From Intrinsic to Switchable Inorganic Antibacterial Surfaces. Eur. J. Inorg. Chem. 2018, 2018 (45), 4846–4855. 10.1002/ejic.201800709.

[ref74] Doveri L.; Taglietti A.; Grisoli P.; Pallavicini P.; Dacarro G. Dual Mode Antibacterial Surfaces Based on Prussian Blue and Silver Nanoparticles. Dalt. Trans. 2023, 52, 452–460. 10.1039/D2DT03058F. 36525102

[ref75] Pallavicini P.; Taglietti A.; Dacarro G.; Antonio Diaz-Fernandez Y.; Galli M.; Grisoli P.; Patrini M.; Santucci De Magistris G.; Zanoni R. Self-Assembled Monolayers of Silver Nanoparticles Firmly Grafted on Glass Surfaces: Low Ag+ Release for an Efficient Antibacterial Activity. J. Colloid Interface Sci. 2010, 350 (1), 110–116. 10.1016/j.jcis.2010.06.019. 20621306

[ref76] Pallavicini P.; de Vita L.; Merlin F.; Milanese C.; Borzenkov M.; Taglietti A.; Chirico G. Suitable Polymeric Coatings to Avoid Localized Surface Plasmon Resonance Hybridization in Printed Patterns of Photothermally Responsive Gold Nanoinks. Molecules 2020, 25 ( (11), ), 2499 10.3390/molecules25112499. 32471310 PMC7321298

[ref77] Jiang S.; Cui C.; Bai W.; Wang W.; Ren E.; Xiao H.; Zhou M.; Cheng C.; Guo R. Shape-Controlled Silver Nanoplates Colored Fabric with Tunable Colors, Photothermal Antibacterial and Colorimetric Detection of Hydrogen Sulfide. J. Colloid Interface Sci. 2022, 626, 1051–1061. 10.1016/j.jcis.2022.07.011. 35868195

[ref78] Liu J.; Sipponen M. H. Ag-Lignin Hybrid Nanoparticles for High-Performance Solar Absorption in Photothermal Antibacterial Chitosan Films. iScience 2023, 26 (10), 108058 10.1016/j.isci.2023.108058. 37854692 PMC10579425

[ref79] Zannotti M.; Vicomandi V.; Rossi A.; Minicucci M.; Ferraro S.; Petetta L.; Giovannetti R. Tuning of Hydrogen Peroxide Etching during the Synthesis of Silver Nanoparticles. An Application of Triangular Nanoplates as Plasmon Sensors for Hg2+ in Aqueous Solution. J. Mol. Liq. 2020, 309, 113238 10.1016/j.molliq.2020.113238.

[ref80] Rehal R. K.; Manoswini M.; Panda A.; Majumdar A. G.; Mahapatra P. K.; Mohanty P. S. Color-Tunable Silver Nanoparticles: Synthesis, Characterizations, and Its Antimicrobial Activity. Bionanoscience 2023, 13 (4), 2448–2457. 10.1007/s12668-023-01127-9.

[ref81] MacPhee J.; Kinyenye T.; MacLean B. J.; Bertin E.; Hallett-Tapley G. L. Investigating the Photothermal Disinfecting Properties of Light-Activated Silver Nanoparticles. Ind. Eng. Chem. Res. 2021, 60 (48), 17390–17398. 10.1021/acs.iecr.1c03165.

[ref82] Liu Y.; Li F.; Guo Z.; Xiao Y.; Zhang Y.; Sun X.; Zhe T.; Cao Y.; Wang L.; Lu Q.; Wang J. Silver Nanoparticle-Embedded Hydrogel as a Photothermal Platform for Combating Bacterial Infections. Chem. Eng. J. 2020, 382, 122990 10.1016/j.cej.2019.122990.

[ref83] Jaswal T.; Gupta J. A Review on the Toxicity of Silver Nanoparticles on Human Health. Mater. Today Proc. 2023, 81 (2), 859–863. 10.1016/j.matpr.2021.04.266.

[ref84] Coughlan C.; Ibáñez M.; Dobrozhan O.; Singh A.; Cabot A.; Ryan K. M. Compound Copper Chalcogenide Nanocrystals. Chem. Rev. 2017, 117 (9), 5865–6109. 10.1021/acs.chemrev.6b00376. 28394585

[ref85] Liu Y.; Liu M.; Swihart M. T. Plasmonic Copper Sulfide-Based Materials: A Brief Introduction to Their Synthesis, Doping, Alloying, and Applications. J. Phys. Chem. C 2017, 121 (25), 13435–13447. 10.1021/acs.jpcc.7b00894.

[ref86] Tang Y.; Qin Z.; Yin S.; Sun H. Transition Metal Oxide and Chalcogenide-Based Nanomaterials for Antibacterial Activities: An Overview. Nanoscale 2021, 13 (13), 6373–6388. 10.1039/D1NR00664A. 33885521

[ref87] Gargioni C.; Borzenkov M.; D’alfonso L.; Sperandeo P.; Polissi A.; Cucca L.; Dacarro G.; Grisoli P.; Pallavicini P.; D’agostino A.; Taglietti A. Self-Assembled Monolayers of Copper Sulfide Nanoparticles on Glass as Antibacterial Coatings. Nanomaterials 2020, 10 ( (2), ), 352 10.3390/nano10020352. 32085548 PMC7075189

[ref88] Chen L.; Hu H.; Chen Y.; Gao J.; Li G. Plasmonic Cu2-XS Nanoparticles: A Brief Introduction of Optical Properties and Applications. Mater. Adv. 2021, 2 (3), 907–926. 10.1039/D0MA00837K.

[ref89] Gu Y.; Jiang Y.; Chen J.; Gao C.; Feng L.; Wu J.; Zhao L. Circular Dichroism and Enantio-Selective Catalytic Effect of Copper Sulfide. Opt. Mater. (Amst). 2022, 132, 112787 10.1016/j.optmat.2022.112787.

[ref90] Nabgan W.; Alqaraghuli H.; Owgi A. H. K.; Ikram M.; Vo D.-V. N.; Jalil A. A.; Djellabi R.; Nordin A. H.; Medina F. A Review on the Design of Nanostructure-Based Materials for Photoelectrochemical Hydrogen Generation from Wastewater: Bibliometric Analysis, Mechanisms, Prospective, and Challenges. Int. J. Hydrogen Energy 2024, 52, 622–663. 10.1016/j.ijhydene.2023.05.152.

[ref91] Goel S.; Chen F.; Cai W. Synthesis and Biomedical Applications of Copper Sulfide Nanoparticles: From Sensors to Theranostics. Small 2014, 10 (4), 631–645. 10.1002/smll.201301174. 24106015 PMC3960363

[ref92] Li M.; Liu X.; Tan L.; Cui Z.; Yang X.; Li Z.; Zheng Y.; Yeung K. W. K.; Chu P. K.; Wu S. Noninvasive Rapid Bacteria-Killing and Acceleration of Wound Healing through Photothermal/Photodynamic/Copper Ion Synergistic Action of a Hybrid Hydrogel. Biomater. Sci. 2018, 6 (8), 2110–2121. 10.1039/C8BM00499D. 29882566

[ref93] Ren Y.; Yan B.; Lin C.; Wang P.; Zhou M.; Cui L.; Yu Y.; Wang Q. Multifunctional Textile Constructed via Polyaniline-Mediated Copper Sulfide Nanoparticle Growth for Rapid Photothermal Antibacterial and Antioxidation Applications. ACS Appl. Nano Mater. 2023, 6, 1212 10.1021/acsanm.2c04797.

[ref94] Huang X.; Hu N.; Wang X.; Zhang Y. S.; Sun R. Copper Sulfide Nanoparticle/Cellulose Composite Paper: Room-Temperature Green Fabrication for NIR Laser-Inducible Ablation of Pathogenic Microorganisms. ACS Sustain. Chem. Eng. 2017, 5 (3), 2648–2655. 10.1021/acssuschemeng.6b03003.

[ref95] Tao B.; Lin C.; Deng Y.; Yuan Z.; Shen X.; Chen M.; He Y.; Peng Z.; Hu Y.; Cai K. Copper-Nanoparticle-Embedded Hydrogel for Killing Bacteria and Promoting Wound Healing with Photothermal Therapy. J. Mater. Chem. B 2019, 7 (15), 2534–2548. 10.1039/C8TB03272F. 32255130

[ref96] Chen S.; Tang F.; Tang L.; Li L. Synthesis of Cu-Nanoparticle Hydrogel with Self-Healing and Photothermal Properties. ACS Appl. Mater. Interfaces 2017, 9 (24), 20895–20903. 10.1021/acsami.7b04956. 28569057

[ref97] Xie Y.; Gan C.; Li Z.; Liu W.; Yang D.; Qiu X. Fabrication of a Lignin-Copper Sulfide-Incorporated PVA Hydrogel with Near-Infrared-Activated Photothermal/Photodynamic/Peroxidase-like Performance for Combating Bacteria and Biofilms. ACS Biomater. Sci. Eng. 2022, 8 (2), 560–569. 10.1021/acsbiomaterials.1c01406. 35077128

[ref98] Shen H.; Zhang C.; Meng Y.; Qiao Y.; Ma Y.; Chen J.; Wang X.; Pan L. Biomimetic Hydrogel Containing Copper Sulfide Nanoparticles and Deferoxamine for Photothermal Therapy of Infected Diabetic Wounds. Adv. Healthc. Mater. 2024, 13 (8), 2303000 10.1002/adhm.202303000. 38063809

[ref99] Wang X. M.; Huang L.; Wang Y. J.; Xuan L.; Li W. W.; Tian L. J. Highly Efficient Near-Infrared Photothermal Antibacterial Membrane with Incorporated Biogenic CuSe Nanoparticles. Chem. Eng. J. 2021, 405, 126711 10.1016/j.cej.2020.126711.

[ref100] Cheng C.; Bao D.; Sun S.; Zhou Y.; Tian L.; Zhang B.; Yu Y.; Guo J.; Zhang S. Chitosan/Copper Sulfide Nanoparticles (CS/CuSNPs) Hybrid Fibers with Improved Mechanical and Photo-Thermal Conversion Properties via Tuning CuSNPs’ Morphological Structures. Int. J. Biol. Macromol. 2023, 253 (P5), 127098 10.1016/j.ijbiomac.2023.127098. 37769777

[ref101] Liu Z.; Bao D.; Jia S.; Qiao J.; Xiang D.; Li H.; Tian L.; Zhang B.; Zhang X.; Zhang H.; Guo J.; Zhang S. The Regulation of CuSNPs’ Interface for Further Enhancing Mechanical and Photothermal Conversion Properties of Chitosan/@CuSNPs Hybrid Fibers. Int. J. Biol. Macromol. 2024, 265 (P1), 130931 10.1016/j.ijbiomac.2024.130931. 38508563

[ref102] Park E.; Selvaraj R.; Kim Y. High-Efficiency Photothermal Sterilization on PDMS Film with Au@CuS Yolk-Shell Nanoparticles. J. Ind. Eng. Chem. 2022, 113, 522–529. 10.1016/j.jiec.2022.06.029.

[ref103] Ren Q.; Yu N.; Zou P.; He Q.; Macharia D. K.; Sheng Y.; Zhu B.; Lin Y.; Wu G.; Chen Z. Reusable Cu2-XS-Modified Masks with Infrared Lamp-Driven Antibacterial and Antiviral Activity for Real-Time Personal Protection. Chem. Eng. J. 2022, 441, 136043 10.1016/j.cej.2022.136043. 35370448 PMC8956354

[ref104] Ren Y.; Yan B.; Lin C.; Wang P.; Zhou M.; Cui L.; Yu Y.; Wang Q. Multifunctional Textile Constructed via Polyaniline-Mediated Copper Sulfide Nanoparticle Growth for Rapid Photothermal Antibacterial and Antioxidation Applications. ACS Appl. Nano Mater. 2023, 6 (2), 1212–1223. 10.1021/acsanm.2c04797.

[ref105] Wang W.; Cao L.; Li Q.; Du C.; Chen S. Copper Sulfide Anchored MXene Improving Photo-Responsive Self-Healing Polyurethane with Enhanced Mechanical and Antibacterial Properties. J. Colloid Interface Sci. 2023, 630, 511–522. 10.1016/j.jcis.2022.10.089. 36334487

[ref106] Qi F.; Li H.; Chen G.; Peng S.; Luo X.; Xiong S.; Zhu H.; Shuai C. A CuS@g-C3N4 Heterojunction Endows Scaffold with Synergetic Antibacterial Effect. Colloids Surfaces B Biointerfaces 2023, 230, 113512 10.1016/j.colsurfb.2023.113512. 37595378

[ref107] Li Q. L.; Sun Y.; Ren L.; Wang X.; Wang C.; Li L.; Yang Y. W.; Yu X.; Yu J. Supramolecular Nanosystem Based on Pillararene-Capped CuS Nanoparticles for Targeted Chemo-Photothermal Therapy. ACS Appl. Mater. Interfaces 2018, 10 (35), 29314–29324. 10.1021/acsami.8b09330. 30091897

[ref108] Sarfraz J.; Borzenkov M.; Niemelä E.; Weinberger C.; Törngren B.; Rosqvist E.; Collini M.; Pallavicini P.; Eriksson J.; Peltonen J.; Ihalainen P.; Chirico G. Photo-Thermal and Cytotoxic Properties of Inkjet-Printed Copper Sulfide Films on Biocompatible Latex Coated Substrates. Appl. Surf. Sci. 2018, 435, 1087–1095. 10.1016/j.apsusc.2017.11.203.

[ref109] Ayaz Ahmed K. B.; Anbazhagan V. Synthesis of Copper Sulfide Nanoparticles and Evaluation of in Vitro Antibacterial Activity and in Vivo Therapeutic Effect in Bacteria-Infected Zebrafish. RSC Adv. 2017, 7 (58), 36644–36652. 10.1039/C7RA05636B.

[ref110] FDA Approves First New Drug Application for Treatment of Radiation Contamination Due to Cesium or Thallium (10/2/2003).

[ref111] Busquets M. A.; Estelrich J. Prussian Blue Nanoparticles: Synthesis, Surface Modification, and Biomedical Applications. Drug Discovery Today 2020, 25 (8), 1431–1443. 10.1016/j.drudis.2020.05.014. 32492486

[ref112] Dacarro G.; Grisoli P.; Borzenkov M.; Milanese C.; Fratini E.; Ferraro G.; Taglietti A.; Pallavicini P. Self-Assembled Monolayers of Prussian Blue Nanoparticles with Photothermal Effect. Supramol. Chem. 2017, 29 (11), 823–833. 10.1080/10610278.2017.1372582.

[ref113] Ngo G.; Félix G.; Dorandeu C.; Devoisselle J.-M.; Costa L.; Milhiet P.-E.; Guari Y.; Larionova J.; Chopineau J. A Novel Approach to the Facile Growth and Organization of Photothermal Prussian Blue Nanocrystals on Different Surfaces. Nanomaterials 2021, 11 (7), 1749 10.3390/nano11071749. 34361135 PMC8308188

[ref114] He X.; Wu H. J.; Wang Y.; Xiang Y.; Zhang K.; Rao X.; Kang E. T.; Xu L. Bimodal Antimicrobial Surfaces of Phytic Acid-Prussian Blue Nanoparticles-Cationic Polymer Networks. Adv. Sci. 2023, 10, 2300354 10.1002/advs.202300354. PMC10238204 37026671

[ref115] Borzenkov M.; D’Alfonso L.; Polissi A.; Sperandeo P.; Collini M.; Dacarro G.; Taglietti A.; Chirico G.; Pallavicini P. Novel Photo-Thermally Active Polyvinyl Alcohol-Prussian Blue Nanoparticles Hydrogel Films Capable of Eradicating Bacteria and Mitigating Biofilms. Nanotechnology 2019, 30 (29), 295702 10.1088/1361-6528/ab15f9. 31025630

[ref116] Wang W.; Ding D.; Zhou K.; Zhang M.; Zhang W.; Yan F.; Cheng N. Prussian Blue and Collagen Loaded Chitosan Nanofibers with NIR-Controlled NO Release and Photothermal Activities for Wound Healing. J. Mater. Sci. Technol. 2021, 93, 17–27. 10.1016/j.jmst.2021.03.037.

[ref117] Kang S.; Pinault M.; Pfefferle L. D.; Elimelech M.; Engineering C.; V Y. U.; Box P. O. Single-Walled Carbon Nanotubes Exhibit Strong Antimicrobial Activity. Langmuir 2007, 23 ( (17), ), 8670–8673 10.1021/la701067r. 17658863

[ref118] Wu C.-S. Antibacterial and Static Dissipating Composites of Poly(Butylene Adipate-Co-Terephthalate) and Multi-Walled Carbon Nanotubes. Carbon N. Y. 2009, 47 (13), 3091–3098. 10.1016/j.carbon.2009.07.023.

[ref119] Chen H.; Wang B.; Gao D.; Guan M.; Zheng L.; Ouyang H.; Chai Z.; Zhao Y.; Feng W. Broad Spectrum Antibacterial Activity of Carbon Nanotubes to Human Gut Bacteria. Small 2013, 9 (16), 2735–2746. 10.1002/smll.201202792. 23463684

[ref120] Lyon D. Y.; Adams L. K.; Falkner J. C.; Alvarez P. J. J. Antibacterial Activity of Fullerene Water Suspensions: Effects of Preparation Method and Particle Size. Environ. Sci. Technol. 2006, 40 (14), 4360–4366. 10.1021/es0603655. 16903271

[ref121] Liu S.; Zeng T. H.; Hofmann M.; Burcombe E.; Wei J.; Jiang R.; Kong J.; Chen Y. Antibacterial Activity of Graphite, Graphite Oxide, Graphene Oxide, and Reduced Graphene Oxide: Membrane and Oxidative Stress. ACS Nano 2011, 5 (9), 6971–6980. 10.1021/nn202451x. 21851105

[ref122] Kang S.; Mauter M. S.; Elimelech M. Microbial Cytotoxicity of Carbon-Based Nanomaterials: Implications for River Water and Wastewater Effluent. Environ. Sci. Technol. 2009, 43 (7), 2648–2653. 10.1021/es8031506. 19452930

[ref123] Xin Q.; Shah H.; Nawaz A.; Xie W.; Akram M. Z.; Batool A.; Tian L.; Jan S. U.; Boddula R.; Guo B.; Liu Q.; Gong J. R. Antibacterial Carbon Based Nanomaterials. Adv. Mater.. 2019, 31 ( (45), ), 1804838 10.1002/adma.201804838. 30379355

[ref124] Jaque D.; Martínez Maestro L.; del Rosal B.; Haro-Gonzalez P.; Benayas A.; Plaza J. L.; Martín Rodríguez E.; García Solé J. Nanoparticles for Photothermal Therapies. Nanoscale 2014, 6, 9494–9530 10.1039/c4nr00708e. 25030381

[ref125] Suo L.; Li Z.; Luo F.; Chen J.; Jia L.; Wang T.; Pei X.; Wan Q. Effect of Dentin Surface Modification Using Carbon Nanotubes on Dental Bonding and Antibacterial Ability. Dent. Mater. J. 2018, 37 (2), 229–236. 10.4012/dmj.2017-023. 29109338

[ref126] Musico Y. L. F.; Santos C. M.; Dalida M. L. P.; Rodrigues D. F. Surface Modification of Membrane Filters Using Graphene and Graphene Oxide-Based Nanomaterials for Bacterial Inactivation and Removal. ACS Sustain. Chem. Eng. 2014, 2 (7), 1559–1565. 10.1021/sc500044p.

[ref127] Lin Y.; Lu K.; Zhang H.; Zou Y.; Chen H.; Zhang Y.; Yu Q. Multifunctional Coatings Based on Candle Soot with Photothermal Bactericidal Property and Desired Biofunctionality. J. Colloid Interface Sci. 2023, 649, 986–995. 10.1016/j.jcis.2023.06.176. 37392688

[ref128] Lin Y.; Zhang H.; Zou Y.; Lu K.; Li L.; Wu Y.; Cheng J.; Zhang Y.; Chen H.; Yu Q. Superhydrophobic Photothermal Coatings Based on Candle Soot for Prevention of Biofilm Formation. J. Mater. Sci. Technol. 2023, 132, 18–26. 10.1016/j.jmst.2022.06.005.

[ref129] Zhang Q.; Liu X.; Tan L.; Cui Z.; Li Z.; Liang Y.; Zhu S.; Yeung K. W. K.; Zheng Y.; Wu S. An UV to NIR-Driven Platform Based on Red Phosphorus/Graphene Oxide Film for Rapid Microbial Inactivation. Chem. Eng. J. 2020, 383, 123088 10.1016/j.cej.2019.123088.

